# Supplementary material for: Neural rhythms as priors of speech computations
Source: Patterns (N Y). 2026 May 19;7(8):101563. doi: 10.1016/j.patter.2026.101563 (PMC13494615; doi:10.1016/j.patter.2026.101563)
Supplement: Supplementary file 1 [file mmc1.pdf]

Patterns, Volume *7*

Supplemental information

Neural rhythms as priors of speech computations

Nand Chandravadia and Nabil Imam

Supplementary Information

Frequency Range	Damping Range	English	Arabic	Bengali
[1, 5] Hz	[0.1, 80]	**90.79% ± 0.84%**	**84.28% ± 6.08%**	**78.01% ± 5.82%**
[0.1, 20] Hz	[0.1, 80]	89.20% ± 0.88%	77.95% ± 8.05%	74.20% ± 6.89%
[20.1, 40] Hz	[20.1, 40]	24.81% ± 10.60%	0.00% ± 0.00%	4.15% ± 1.77%
[40.1, 60] Hz	[40.1, 60]	2.17% ± 0.90%	0.00% ± 0.00%	0.00% ± 0.00%
[60.1, 80] Hz	[60.1, 80]	0.00% ± 0.00%	0.00% ± 0.00%	0.73% ± 0.60%
[80.1, 160] Hz	[80.1, 160]	0.00% ± 0.00%	0.00% ± 0.00%	0.00% ± 0.00%
[160.1, 240] Hz	[160.1, 240]	0.00% ± 0.00%	0.00% ± 0.00%	0.00% ± 0.00%
[250.1, 330] Hz	[250.1, 330]	0.00% ± 0.00%	0.00% ± 0.00%	0.00% ± 0.00%
[400.1, 480] Hz	[400.1, 480]	0.00% ± 0.00%	0.00% ± 0.00%	0.00% ± 0.00%
[1000.1, 1080] Hz	[1000.1, 1080]	0.00% ± 0.00%	0.00% ± 0.00%	0.00% ± 0.00%
No Rhythm	No Damping	4.90% ± 1.55%	4.80% ± 1.52%	3.01% ± 1.45%

Table S1: Final test accuracy of networks configured with frequency and damping coefficients selected uniformly at random from the specified ranges. The bold values indicate the highest accuracies. Values are averaged (mean ± standard error) across 10 random seeds.

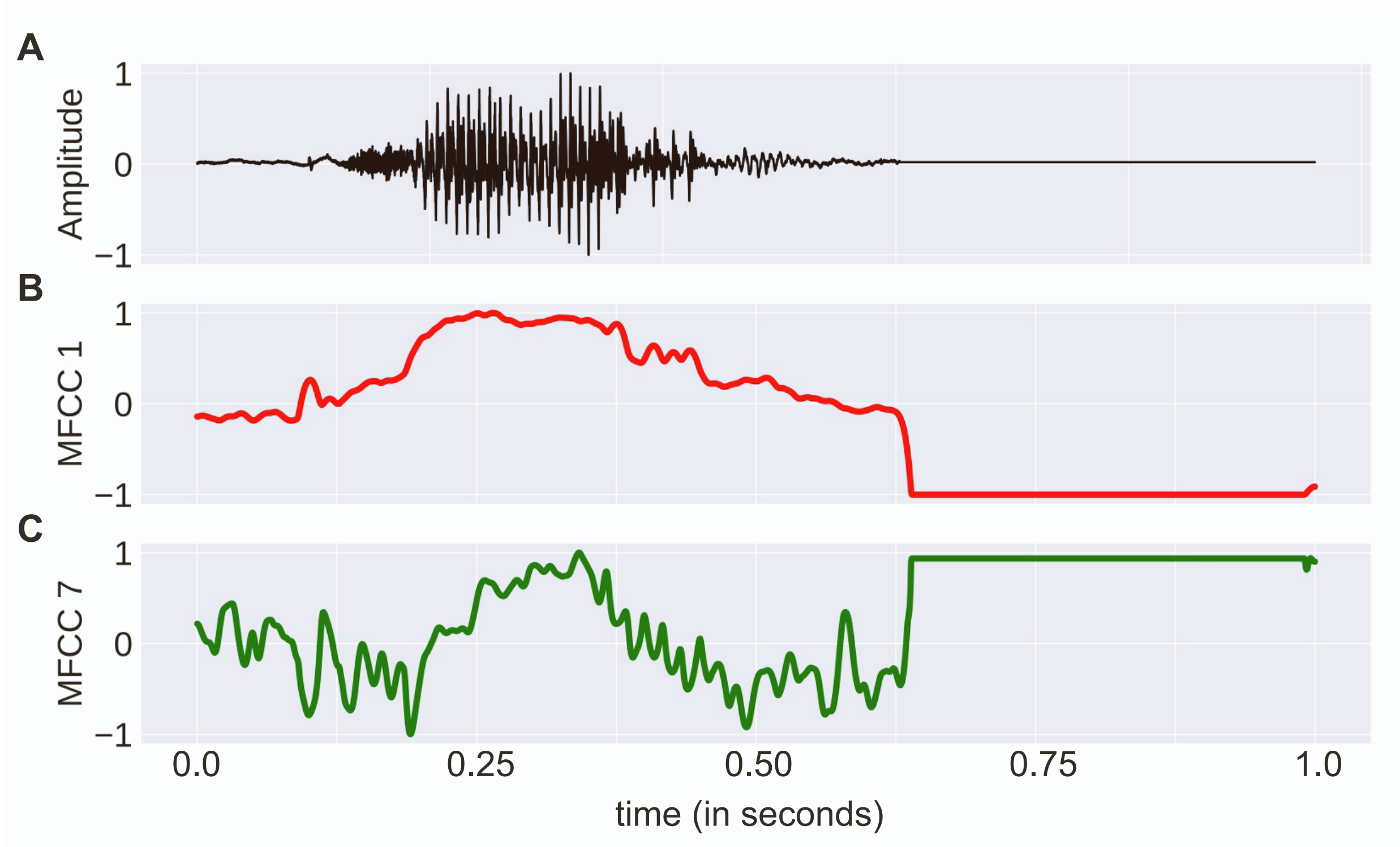

Figure S1: Mel-frequency cepstral coefficients (MFCCs) extracted from raw speech waveform. **(A)** Waveform of the spoken word "zero." **(B)** First MFCC coefficient, which closely resembles the speech envelope. **(C)** Seventh MFCC coefficient, which reflects finer spectral details. All MFCCs were normalized to [-1, 1] for comparison with the raw waveform.

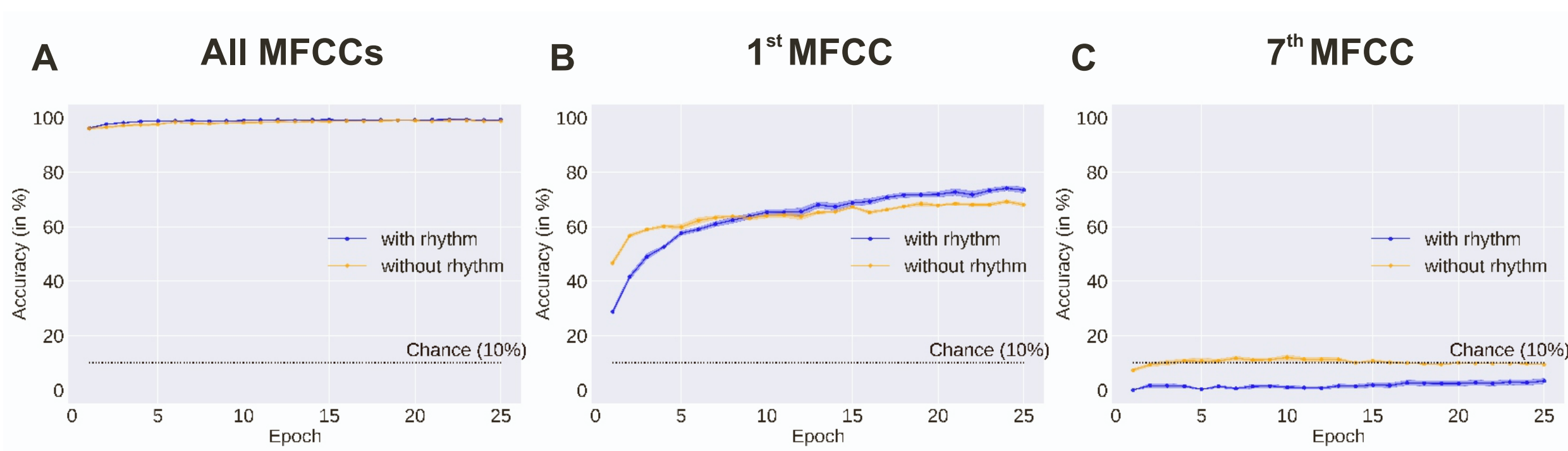

Figure S2: Performance with mel-frequency cepstral coefficients (MFCCs). Test accuracy per epoch of networks configured with frequency coefficients chosen uniformly at random from the range [1 Hz, 3 Hz] (blue). Values are averaged across ten random seeds. Shaded region depicts standard error. For reference, performance of the same network without endogenous oscillations is shown in yellow. Mel coefficients were **(A)** All, **(B)** 1st, **(C)** 7th.

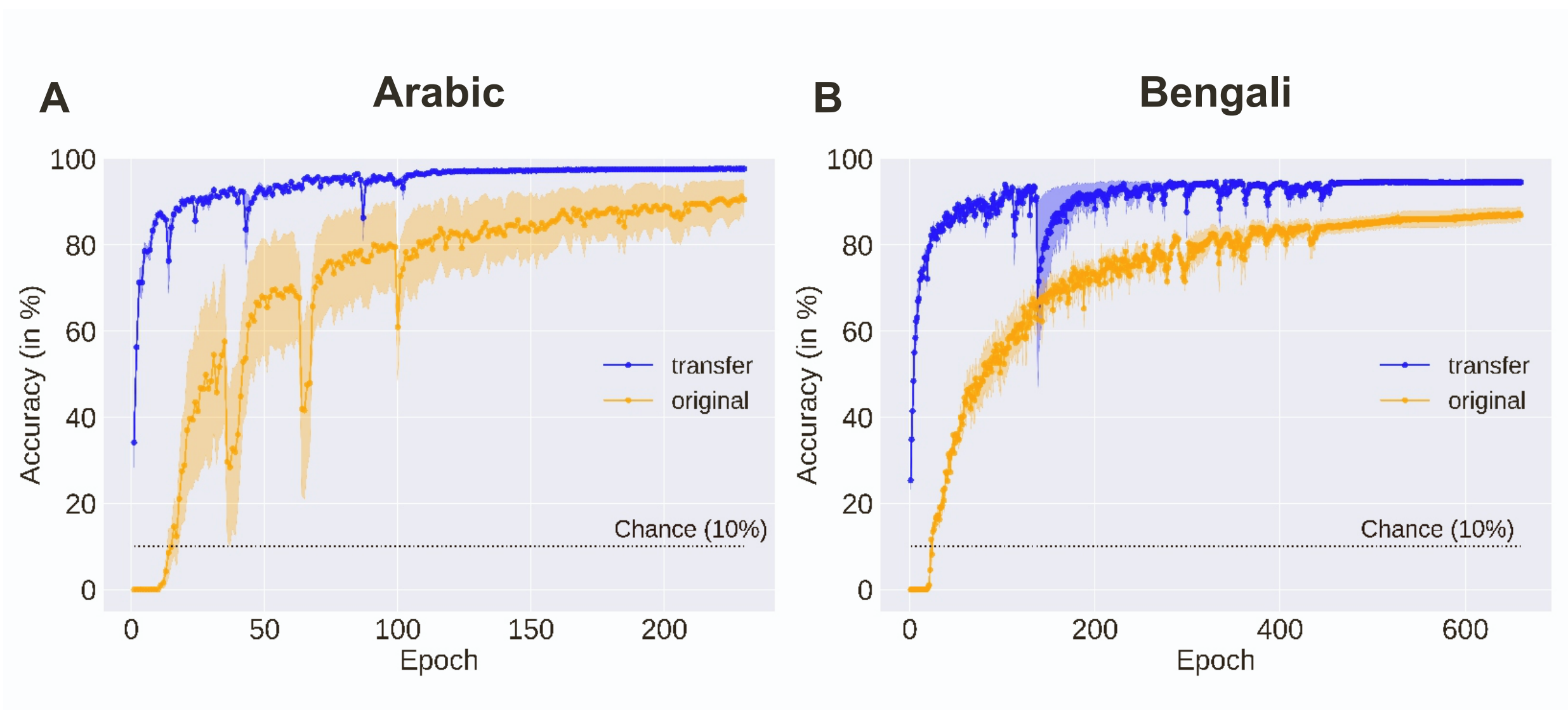

Figure S3: Transfer learning across languages. **(A)** Test accuracy per epoch for Arabic spoken digit recognition, comparing a network initialized with the parameters of a pre-trained English model (blue) to a network trained from scratch (yellow). **(B)** Same setup for Bengali spoken digit recognition. In both cases, the pre-trained network reaches near-perfect accuracy within a few epochs, whereas training from scratch requires over 100 epochs. Values are averaged across three random seeds. Shaded regions depict standard error.

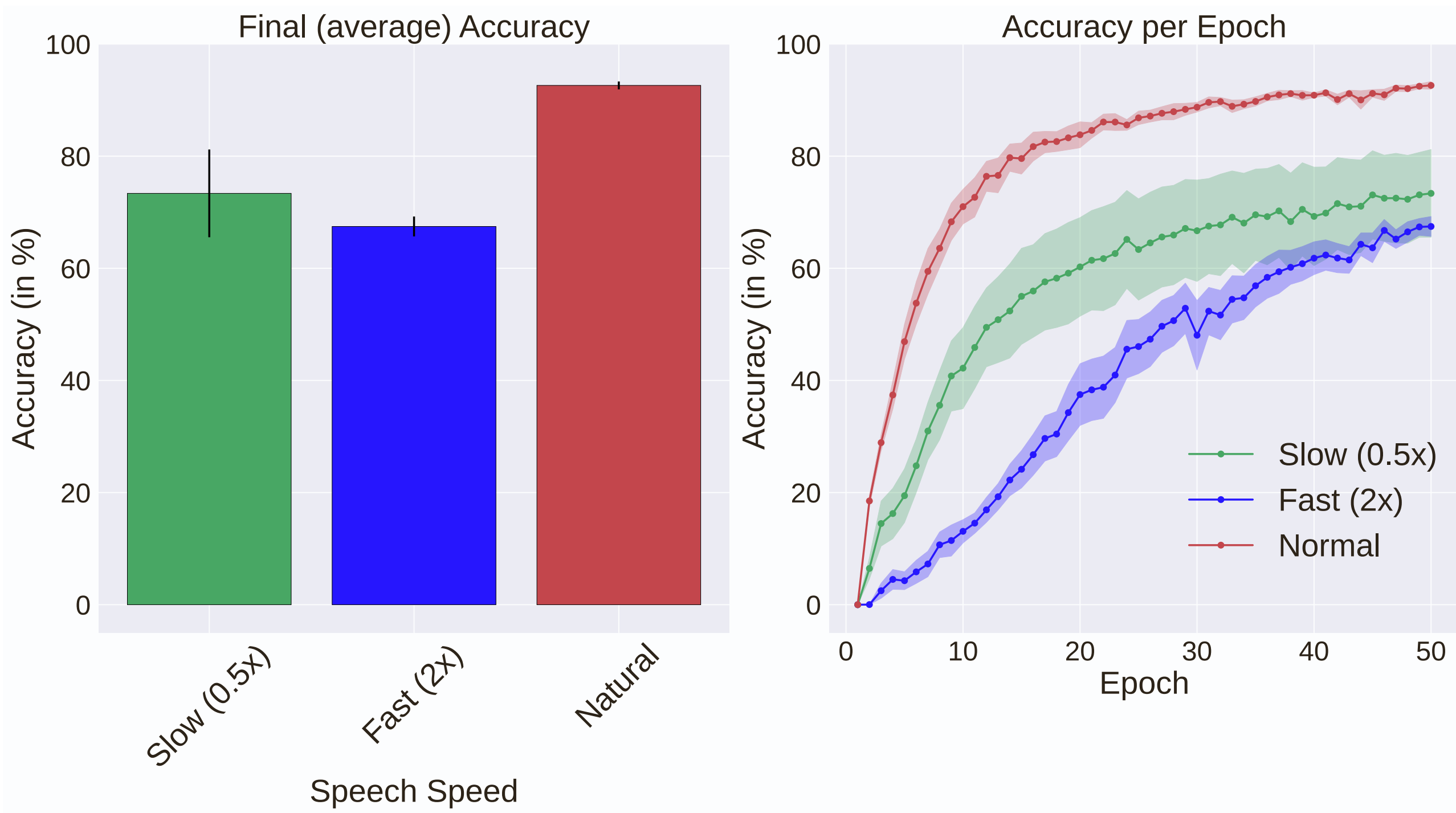

Figure S4: Effect of speech rate manipulation on performance. *Left:* Test accuracy of networks configured with [1 Hz, 5 Hz] frequency coefficients when the speech signal is slowed ($0.5x$), sped up ($2x$), and natural. *Right:* Test accuracy per epoch for the same networks. Values are averaged across ten random seeds. The shaded region depicts standard error.

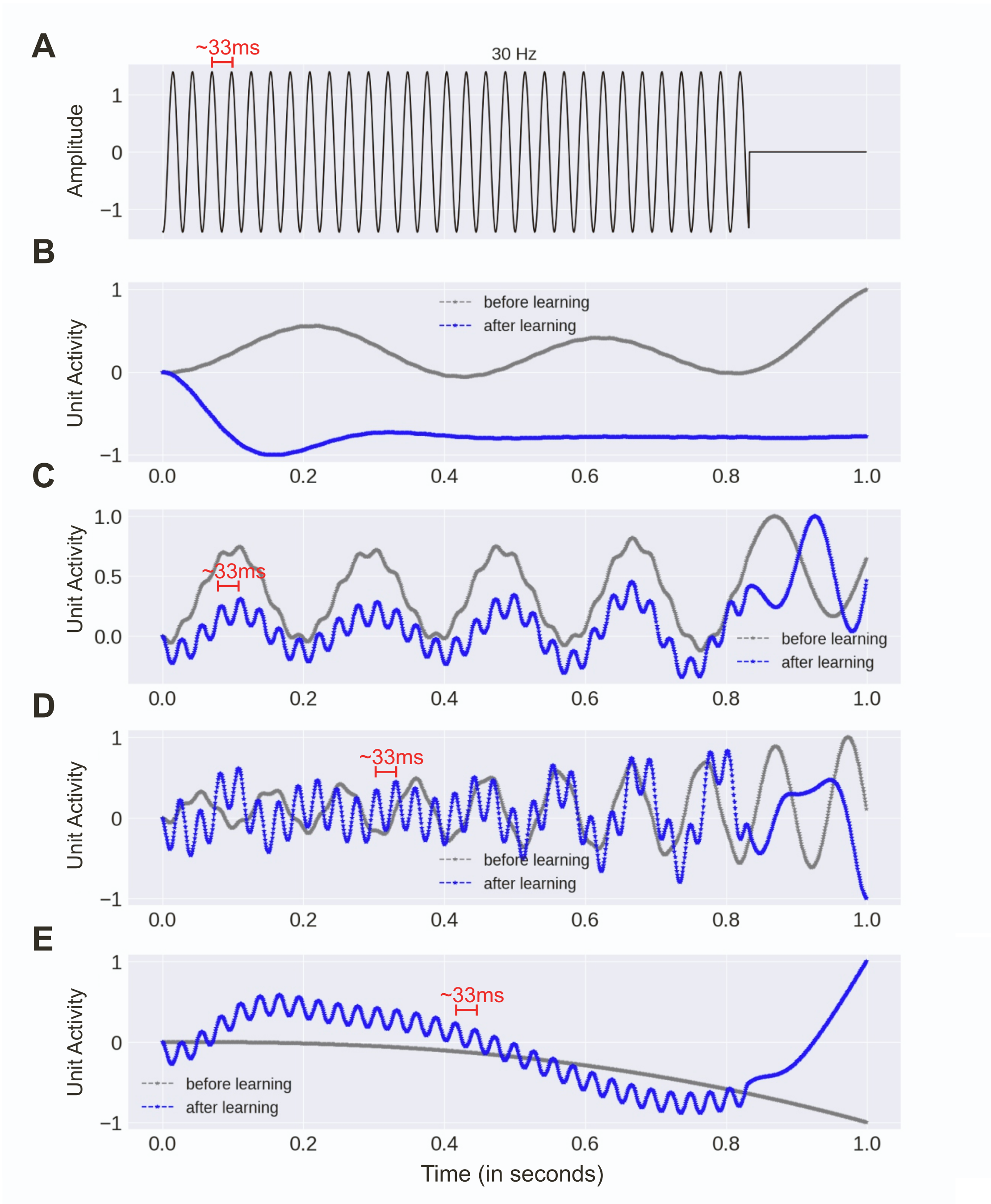

Figure S5: Network oscillations in response to a 30 Hz sinusoidal input. **(A)** Input waveform. **(B)** Response of an oscillator in a network configured with frequency coefficients drawn uniformly at random from the range [1 Hz, 10 Hz], **(C)** [10 Hz, 100 Hz], **(D)** [100 Hz, 110 Hz]. **(E)** Response when the frequency coefficients are set to 0. Oscillator activity is plotted as its normalized amplitude (scaled to [-1,1]). All networks, except for the network in **B**, tracks the temporal modulations of the input and correctly classifies the signal.

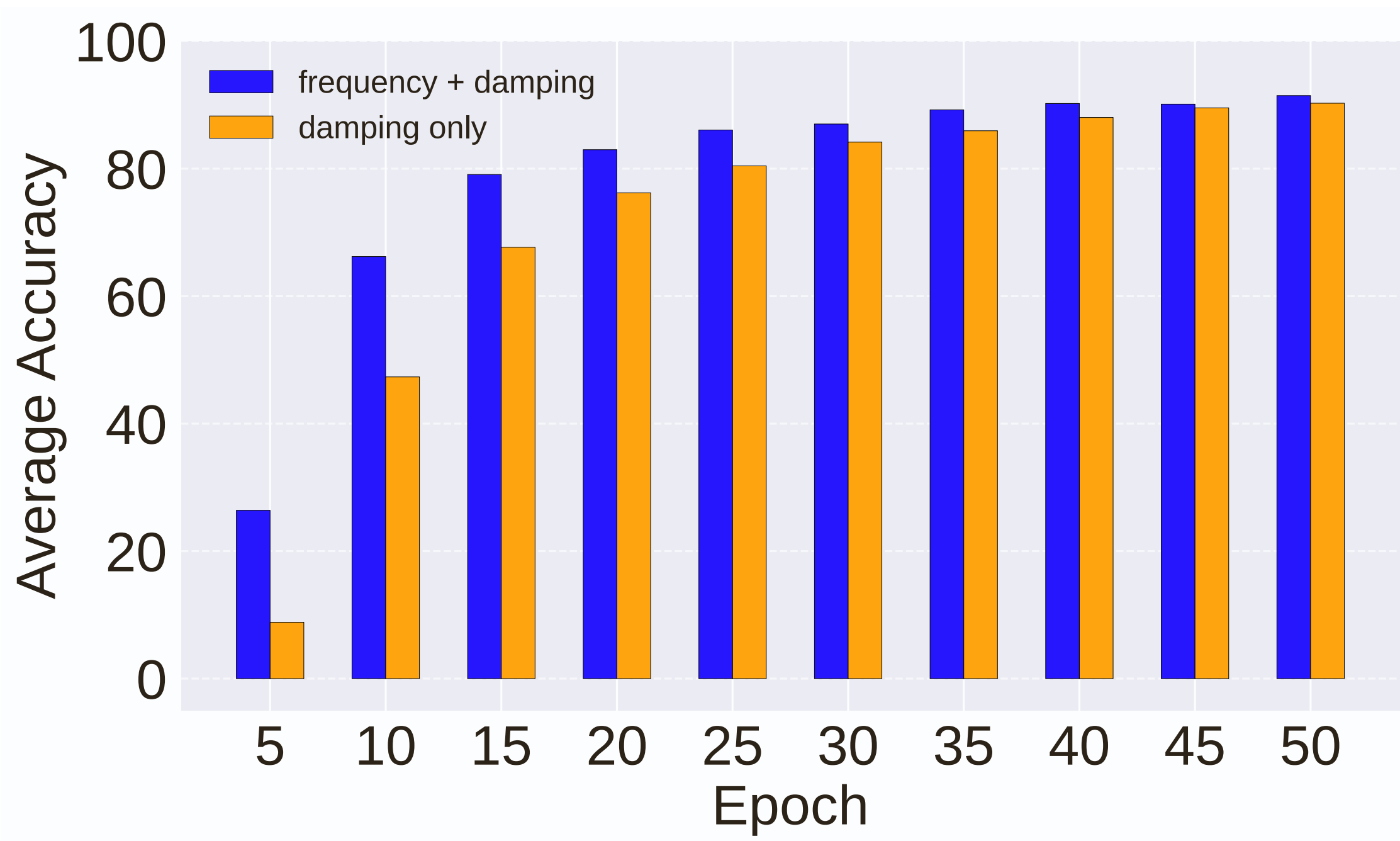

Figure S6: Task accuracy with damping only. Blue: Test accuracy on AudioMNIST (after every five epochs of training) of a network configured with frequency and damping coefficients drawn uniformly at random from the range [1 Hz, 3 Hz] and [0.1, 80], respectively. Yellow: same as blue, but with frequency coefficients set to 0. This network learns at a slower pace but eventually reaches high accuracy once it learns to oscillate with the speech envelope. Performances shown are averaged cross three random seeds.

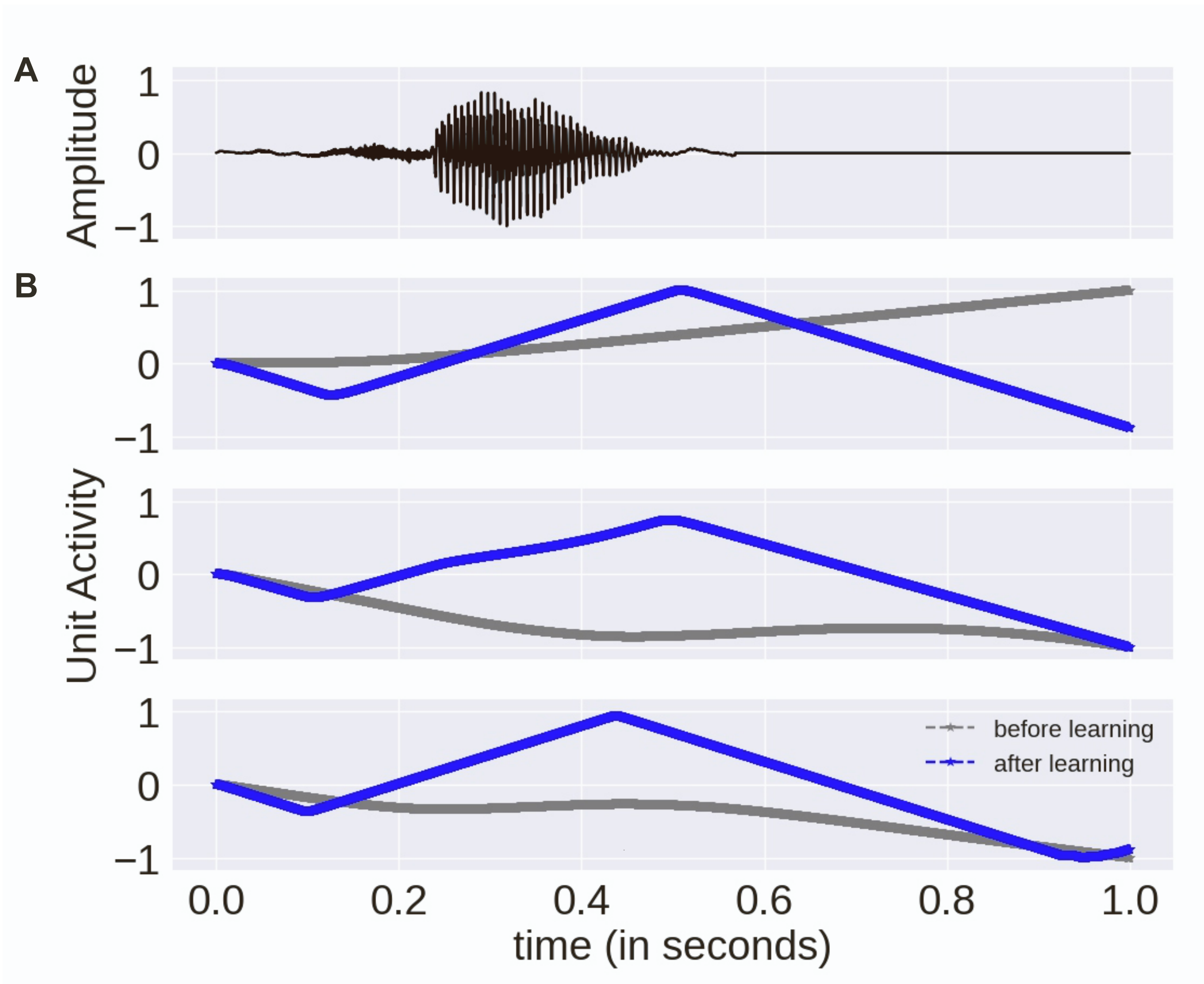

Figure S7: Oscillations in damped networks. **(A)** Waveform of the spoken word "three." **(B)** Example response of three network oscillators before (grey) and after training (blue) on AudioMNIST. The frequency coefficient of the network is set to 0 and the damping coefficients are drawn uniformly at random from the range [0.1, 80]. Before training, no clear oscillatory pattern is present. After training, the oscillators exhibit rhythmic activity at approximately 1 Hz, matching the approximately 1 Hz rhythm of the speech envelope. The network classified the input correctly after training. Oscillator activity is plotted as its normalized amplitude (scaled to [-1, 1]).

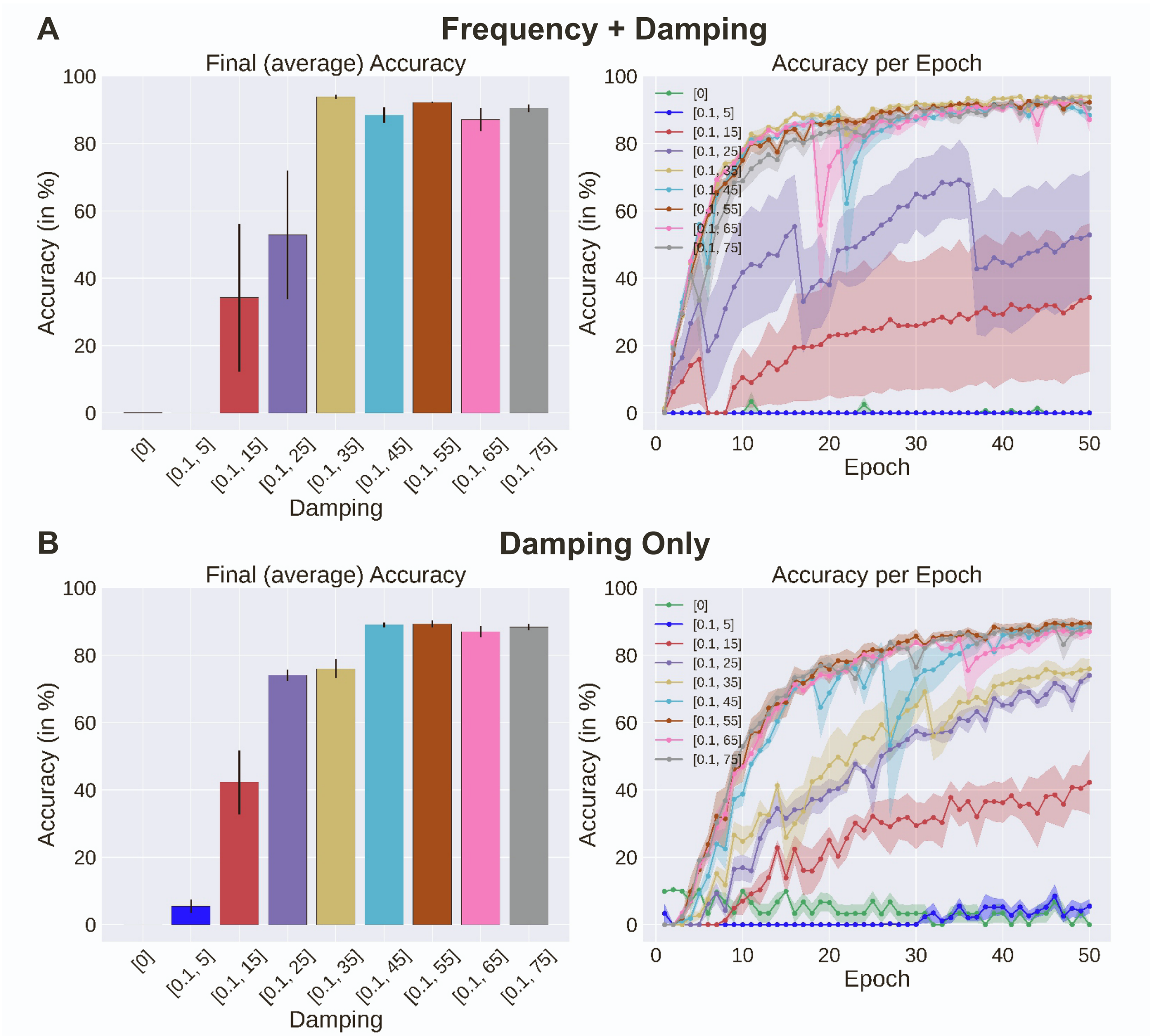

Figure S8: Impact of damping coefficient on network performance. **(A)** Accuracy in detecting English spoken words. Left: Test accuracy of networks configured with damping coefficients drawn uniformly at random from the specified ranges and oscillator frequencies drawn from the range $[1, 3]$ Hz. Values are averaged across three random seeds. Error bars depict standard error. Right: Test accuracy after every epoch of training. **(B)** Performance of networks with the same damping coefficients but with the frequency coefficient set to 0, illustrating a similar dependence on damping.

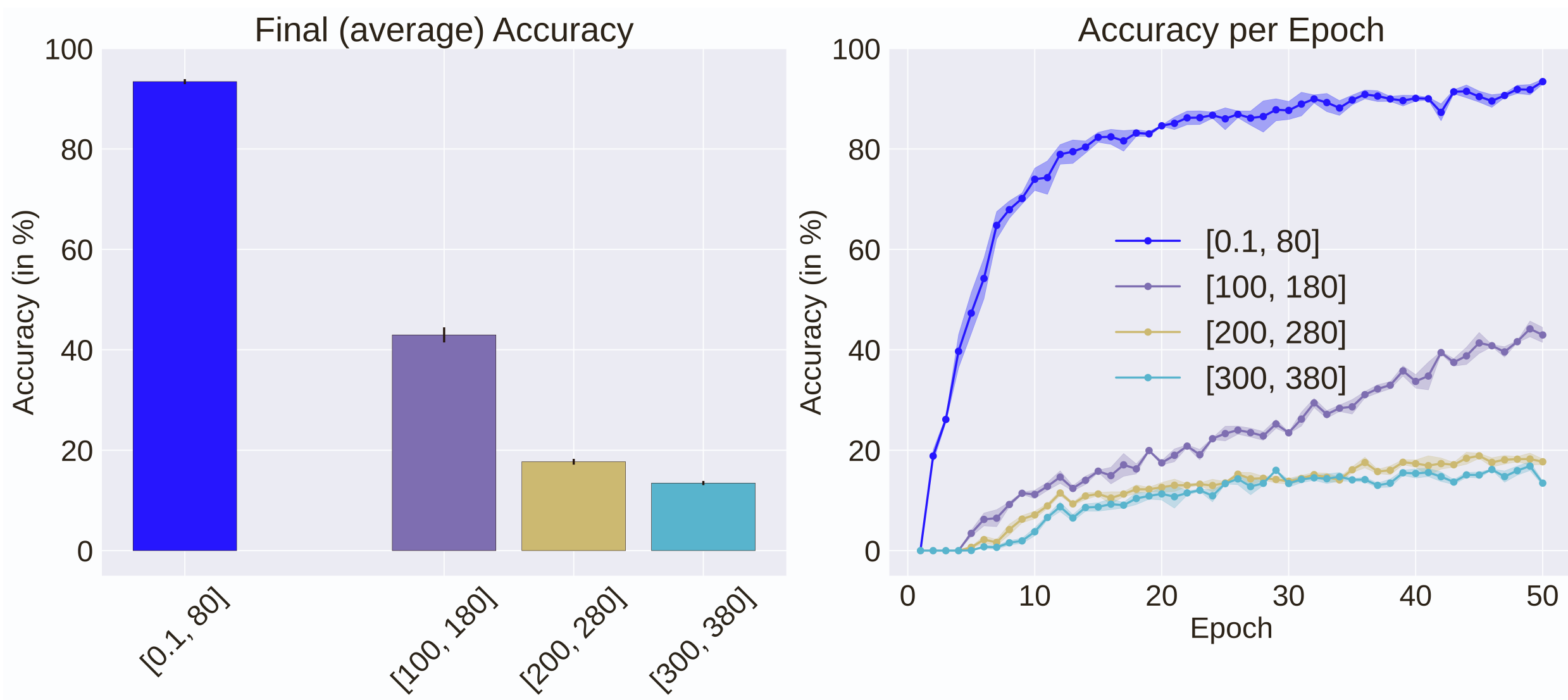

Figure S9: Impact of high damping coefficient on network performance. Accuracy in detecting English spoken words. Left: Test accuracy of networks configured with damping coefficients drawn uniformly at random from the specified ranges and oscillator frequencies drawn from the range $[1, 3]$ Hz. Values are averaged across three random seeds. Error bars depict standard error. Right: Test accuracy after every epoch of training. Overdamping the network substantially reduces performance.

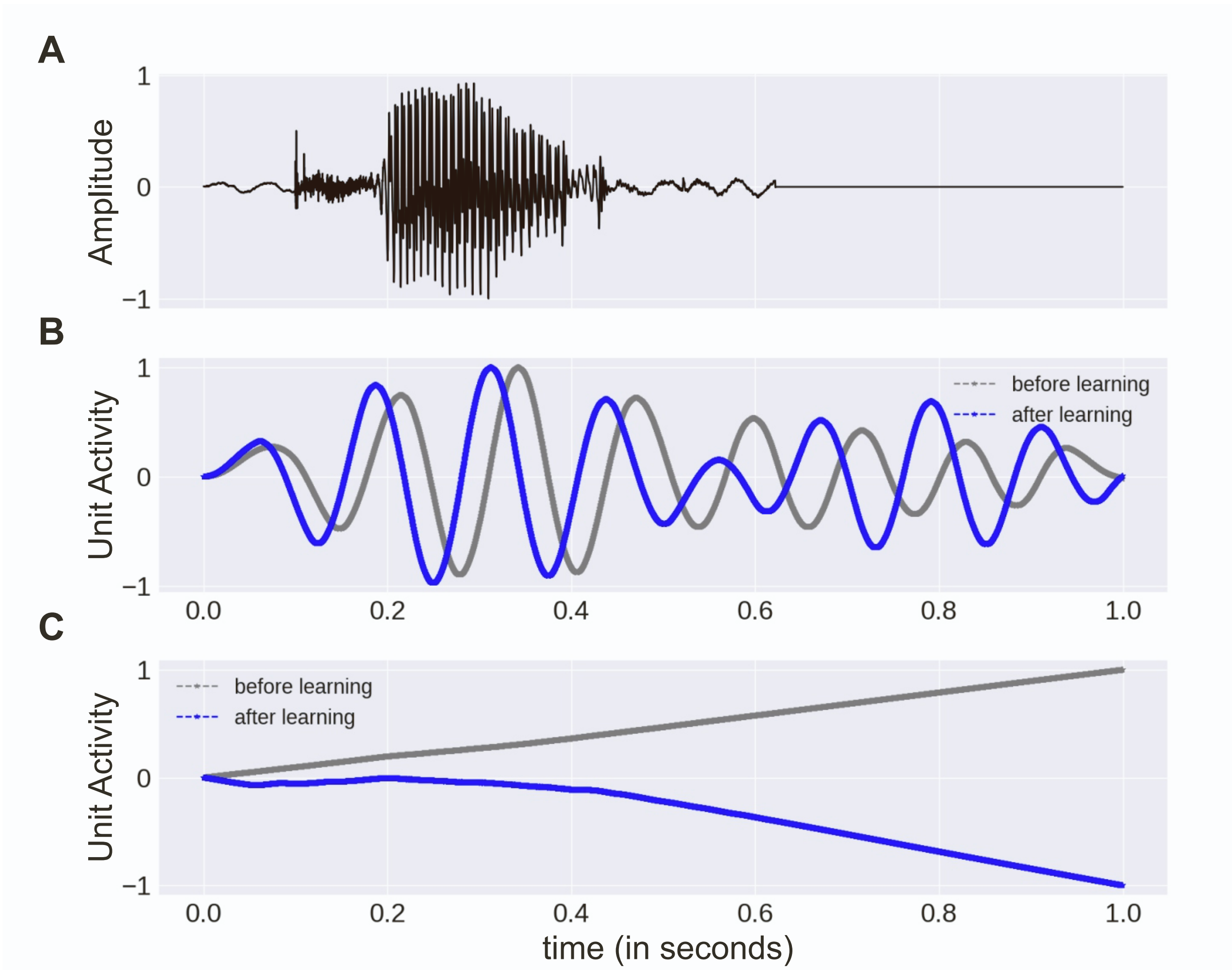

Figure S10: Network oscillations as a result of no damping and excessive damping. **(A)** Waveform of the spoken word "two" from the AudioMNIST dataset. **(B)** Example response of an oscillator in a network with damping coefficients set to 0 and frequency coefficients drawn from the range [1, 3] Hz. **(C)** Example response of an oscillator in a network with very high damping ($\epsilon = 240$) and frequency coefficients drawn from the range [1 Hz, 3 Hz]. Oscillator activity is plotted as its normalized amplitude (scaled to [-1,1]). Both networks classified the input incorrectly after training on the full dataset.
