# Supplementary material for: Neural rhythms as priors of speech computations
Source: Patterns (N Y). 2026 May 19;7(8):101563. doi: 10.1016/j.patter.2026.101563 (PMC13494615; doi:10.1016/j.patter.2026.101563)
Supplement: Supplementary file 2 [file mmc2.pdf]

Article

Patterns

Neural rhythms as priors of speech computations

Highlights

- Speech rhythms and brain rhythms exhibit similar temporal structure
- Embedding brain rhythms into a recurrent neural network improves speech recognition
- Networks with low-frequency rhythms achieve the best performance
- These benefits extend across three natural languages but not to urban sounds

Authors

Nand Chandravadia, Nabil Imam

Correspondence

nchandravadia3@gatech.edu (N.C.),
nimam6@gatech.edu (N.I.)

In brief

Network rhythms are ubiquitous in the brain, but their computational role remains unclear. The authors show that embedding brain-like rhythms into a recurrent neural network significantly improves speech recognition across languages but not recognition of non-speech sounds, suggesting that brain rhythms encode innate knowledge of speech structure.

Chandravadia & Imam, 2026, Patterns 7, 101563
August 14, 2026 © 2026 The Author(s). Published by Elsevier Inc.
https://doi.org/10.1016/j.patter.2026.101563

CellPress

Patterns

CellPress
OPEN ACCESS

Article

Neural rhythms as priors of speech computations

Nand Chandravadia[1,2,*] and Nabil Imam[1,2,3,*]
[1]School of Computational Science and Engineering, Georgia Institute of Technology, Atlanta, GA 30332, USA
[2]Machine Learning Center (ML@GT), Georgia Institute of Technology, Atlanta, GA 30332, USA
[3]Lead contact
*Correspondence: nchandravadia3@gatech.edu (N.C.), nimam6@gatech.edu (N.I.)
https://doi.org/10.1016/j.patter.2026.101563

THE BIGGER PICTURE People speak at particular rhythms, producing syllables, words, and phrases at characteristic rates, and these rhythms help make speech comprehensible despite variability across speakers, accents, and languages. Auditory neural networks in the brain generate rhythms at these same rates, suggesting that brain circuits may be innately tuned to the temporal structure of speech. In an artificial neural network incorporating brain-like rhythms, we find that speech is readily recognized across different speakers and languages if the configured rhythms match the temporal modulations of speech. Without these rhythms, the network is unable to learn meaningful representations and consequently performs at chance levels. This stark difference in recognition performance indicates that wave-like rhythms in the auditory system serve as inductive biases that are exploited for learning speech signals. More broadly, embedding wave dynamics into artificial neural networks offers a new design principle for building machines that learn more efficiently.

SUMMARY

Endogenous rhythms of auditory neural circuits have a striking resemblance to the temporal modulations of incoming speech signals. Here, we show that these rhythms may serve as priors for speech recognition, encoding knowledge of speech structure in the dynamics of network computations. In a network of coupled oscillators, we find that speech is readily identified when characteristic frequencies of the oscillators match low-frequency circuit rhythms in the auditory cortex. When signal and circuit rhythms are mismatched, speech identification is impaired. Compared to a baseline recurrent neural network without intrinsic oscillations, the coupled oscillatory network has significantly higher performance in speech recognition across languages but not in the recognition of signals that lack speech-like structure, such as urban sounds. Our results suggest a central computational role of brain rhythms in speech processing.

INTRODUCTION

Parsing a continuous stream of speech within mixtures of unrelated sounds relies on coordinated neural processes in the brain that track temporal fluctuations of the speech envelope. The auditory cortex displays characteristic patterns of neural activity, notably low-frequency neural oscillations ("rhythms") in the delta (δ) and theta (θ) bands and heightened sensitivity to stimulus modulations in this range.[1–4] Interestingly, temporal modulations of speech envelopes exhibit rhythms in a similar low-frequency range, with envelope spectra typically peaking at around 4 Hz.[5–7] When the envelope is artificially compressed, speech comprehension significantly degrades but recovers when silent gaps are inserted to restore the natural temporal structure of the signal.[8] These observations suggest that the natural frequencies of auditory neural circuits provide a temporal scaffold upon which speech computations take place.

Temporally structured neural activity patterns in the brain tile a range of roughly three orders of magnitude from ultra-slow (<0.01 Hz) to ultra-fast ($\sim$600 Hz) oscillations.[9] Within this broad range are five functional oscillation classes—δ, θ, alpha (α), beta (β), and gamma (γ)—whose interactions are presumed to subserve various functions, including speech and language processing. It has been proposed that networks of inhibitory and excitatory neurons underlying these oscillations give rise to larger computational units that function as coupled harmonic oscillators.[5,10–12] These networks of oscillators exhibit periodic (wave-like) behavior characterized by frequency, amplitude, and phase, with coupling strengths dynamically adjusted in response to external stimuli. Coupling between oscillators operating at different timescales reflects hierarchical temporal information integration in the brain.

Here, we use model networks of globally coupled oscillators[12,13] trained with machine learning tools to investigate the relationship between endogenous network rhythms of the brain

This is an open access article under the CC BY license (http://creativecommons.org/licenses/by/4.0/).

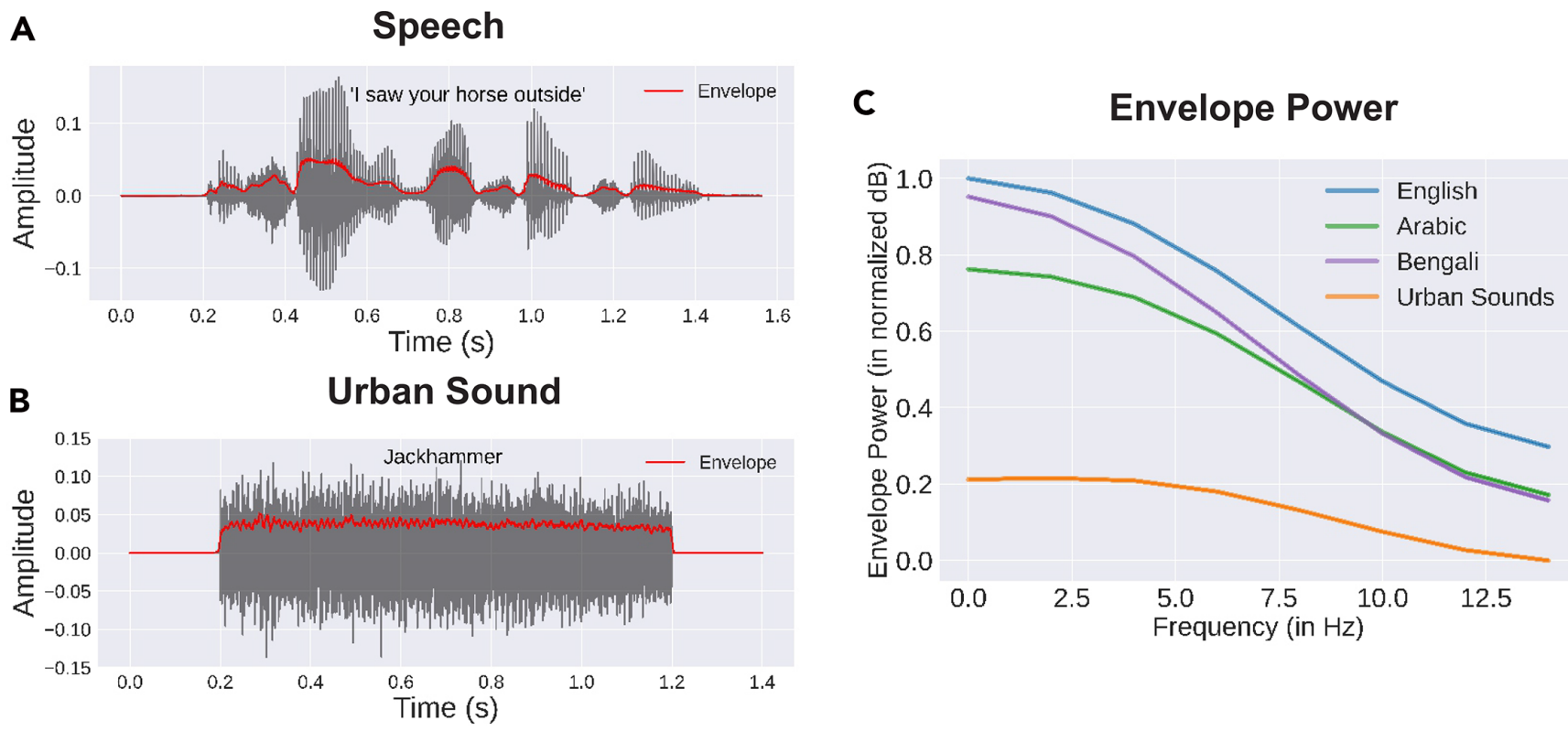

Figure 1. Dynamics of speech and non-speech stimuli

(A) Raw audio of the spoken sentence "I saw your horse outside" (gray) with the corresponding speech envelope (red).

(B) Raw audio of an urban sound ("jackhammer") (gray) and its envelope (red).

(C) Average power (in normalized dB, [0,1]) of the envelopes in three spoken language datasets—English, Arabic, and Bengali—and for a dataset of urban sounds.

and the natural rhythms of input speech signals. The dynamics of each network is defined by the characteristic frequency and damping coefficient of individual oscillators, as well as their coupling strengths, which are learned from speech data. These intrinsic and learned network parameters determine network trajectories over time, which are decoded to categorize speech utterances. We demonstrate that (1) encoding *a priori* low-frequency brain-like rhythms in the oscillators significantly improves speech recognition performance compared to conventional recurrent neural networks (RNNs); (2) these computational benefits are consistent across three languages, English, Arabic, and Bengali; (3) network rhythms that align best with the temporal modulations of the speech envelope provide the greatest performance gains across all speech tasks; and (4) these benefits are absent in a non-speech task involving the recognition of urban sounds that lack the temporal structure of speech. These results suggest that low-frequency rhythms in the auditory cortex serve as a dynamic scaffold for speech processing, where coupled oscillations act through the superposition and interference patterns of waves to efficiently extract structured temporal patterns in speech.

RESULTS

Rhythms of speech

The temporal modulations of the speech envelope have a peak energy concentration between 1 and 5 Hz.[5,14] These modulations reflect the natural phrasal and syllabic rhythms of spoken language (Figure 1A). The envelope, which represents the slow amplitude fluctuations of the speech signal over time, encodes the overall intensity pattern of speech and reflects essential rhythmic features of spoken language, such as syllabic stress and phrasal timing. The hierarchical organization of language is captured in these low-frequency modulations, with slower components conveying phrase-level structure and faster ones conveying syllabic transitions, enabling the segmentation and interpretation of continuous speech.

Unlike the structured and hierarchical rhythms of speech, urban sounds encompass a wide variety of auditory patterns, including irregular noises such as car horns or construction activity (Figure 1B). These sounds lack the temporal regularity and predictable modulations characteristic of speech, instead featuring abrupt onsets, wide spectral distributions, and overlapping frequencies. For instance, while the speech envelope predominantly occupies low-frequency bands between 1 and 5 Hz, urban sounds span a broader range of frequencies with erratic amplitude fluctuations. The envelope of urban sounds reflects these irregularities, with peaks and valleys occurring sporadically rather than in systematic rhythmic patterns.

To assess differences in the modulation spectra of speech and urban sounds, we analyze the envelope power in datasets of spoken words across three languages—English, Arabic, and Bengali—and compare it to that of urban sounds encountered in daily life (Figure 1C). For this, we first compute the amplitude envelope for each sample in the training corpus of each dataset (English, Arabic, Bengali, and urban sounds). Then, we calculate the power spectrum of each envelope and average them to obtain a representative modulation spectrum. Across languages, we observe a distinctive pattern: the speech envelope peaks between the 1–5 Hz frequency range, reflecting the regular rate at which speakers of different languages convey phonetic information (Figure 1C, English, Arabic, and Bengali). In contrast, urban sounds lack a putative rhythmic pattern, exhibiting no clear peak in the power of the envelope (Figure 1C, urban sounds). The dataset for each language consists of spoken words from different speakers, while the dataset for urban sounds consists of common daily sounds, such as a jackhammer and car horn (methods).

Rhythms in RNNs

The regular temporal structure of speech raises the question of whether the auditory system encodes innate structural knowledge of the signal prior to language acquisition. What computational advantages could this provide? To investigate, we construct a network of coupled oscillators (Figure 2) to model population-level activity in the auditory cortex.[5,12–15] Each oscillator is defined by a characteristic frequency (rhythm) and a damping coefficient, which together determine its intrinsic response (methods). The oscillators are simultaneously driven by this intrinsic dynamic and by raw speech input that modulates network activity. The network adopts an all-to-all coupling architecture, in which the activity of each oscillator influences every other unit, including itself. Internal couplings—determining how oscillators influence each other's activity—are initialized randomly but adapt through training to encode stimulus structure. As a result, the network learns a rich and diverse set of

CellPress
OPEN ACCESS

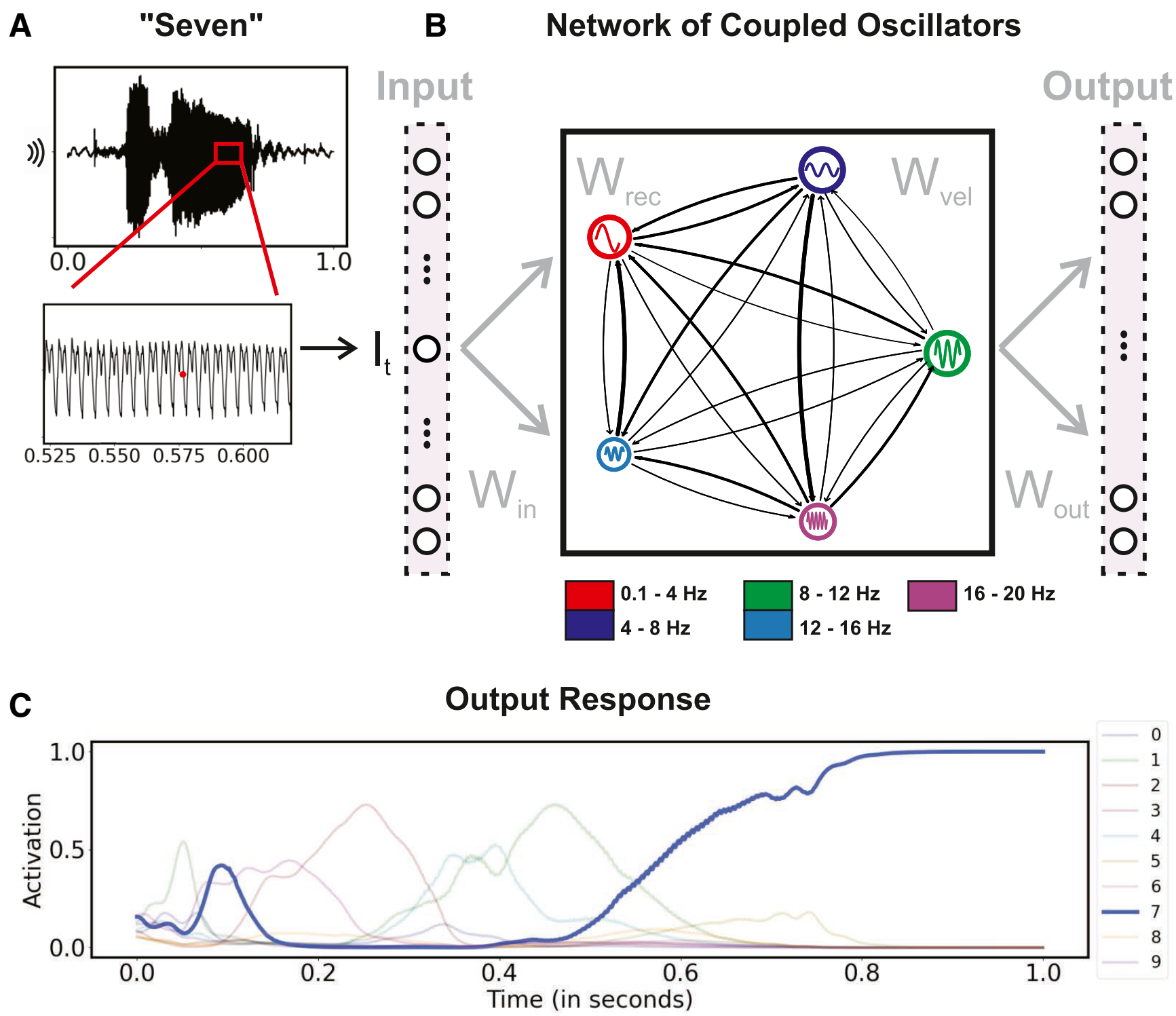

Figure 2. Network architecture

(A) Time-varying speech waveform of the spoken word "seven," sampled at each time point I_t.

(B) Illustration of a network of coupled oscillators receiving samples of the speech waveform, with plastic input (W_{in}), recurrent (W_{rec} and W_{vel}), and output (W_{out}) weights.

(C) Activity in the output layer in response to the spoken word.

trajectories in its state space, shaped by its intrinsic rhythms and the temporal regularities of speech.

We configure the oscillators with heterogeneous frequencies drawn from a fixed range. We evaluate several frequency ranges: some corresponding to brain oscillations (e.g., δ, θ, and γ bands), while others have little correspondence to brain activity. During learning, we present raw auditory waveforms to the network, and the network adapts its coupling parameters so that the waveforms—either speech or non-speech—are classified into appropriate categories (methods). For instance, repeated presentations of the spoken word "seven" guide the network toward a dynamical trajectory from which a linear readout layer determines the stimulus class. Once trained, we present the network with unseen samples of the learned categories and record its recognition performance.

Performance across languages

To assess the effects of endogenous network oscillations on speech processing, we evaluate the performance of our model network in a series of tasks, including spoken word recognition in three different languages and recognition of altered speech and non-speech sounds.

First, we initialize a network of 256 oscillators, with characteristic frequencies drawn uniformly at random from the range 0.1–20 Hz. We train this network on a dataset of spoken English words, consisting of utterances of the words "zero" through "nine" by different speakers. Each spoken word in the training set was uttered multiple times. During training, we feed the network the raw speech waveform (as recorded from the speaker) and adjust the coupling strengths via the backpropagation through time (BPTT) learning algorithm (methods).[16,17] After training the network for several epochs, we evaluate its word recognition performance on a held-out test set. As shown in Figure 3A, the network achieves robust accuracy within the first few epochs, reaching a final performance of 89% ± 1% after 50 epochs. Accuracy values are averaged over 10 random initializations of network parameters. Notably, this high test performance is achieved using only the raw speech waveform as input—without any pre-processing or feature extraction—during both training and testing.

For comparison, we train a RNN without endogenous oscillations on the same dataset of spoken English digits, using the same learning algorithm and raw speech input (methods). Unlike the oscillatory network, this RNN was unable to learn the task (Figure 3A). Test accuracy remained at 5% ± 2% throughout training.

Next, we assess which oscillator frequencies are most effective in recognizing speech. We initialize different networks, each configured with frequencies drawn uniformly at random from a specific range, spanning ranges from around 1 Hz to over 200 Hz. We find that networks with oscillator frequencies in the range 1–5 Hz achieve the highest accuracy and training efficiency (Figure 4A). Interestingly, this corresponds to the peak envelope power of the speech signal (Figure 1C), indicating that a match between signal and network oscillations leads to the best accuracy. Accuracy dropped steeply with higher-frequency network oscillations, falling to chance levels for frequencies above 40 Hz.

These results indicate that the coupled oscillatory network achieves high performance when it encodes knowledge of the temporal structure of speech—specifically, its amplitude modulation frequencies—prior to training. To test whether this effect extends beyond English, we evaluate the network on two additional datasets: Arabic and Bengali Spoken Digits.

The Arabic and Bengali Spoken Digits datasets contain utterances of the digits "zero" through "nine" in Arabic and Bengali, respectively, each spoken multiple times by different speakers. As before, the network is trained using the raw speech waveform without any pre-processing or alignment. The results closely mirror those observed for English. Networks configured with low-frequency oscillations achieve substantially higher performance (78% ± 8% for Arabic and 74% ± 7% for Bengali) compared to networks that lacked endogenous oscillations

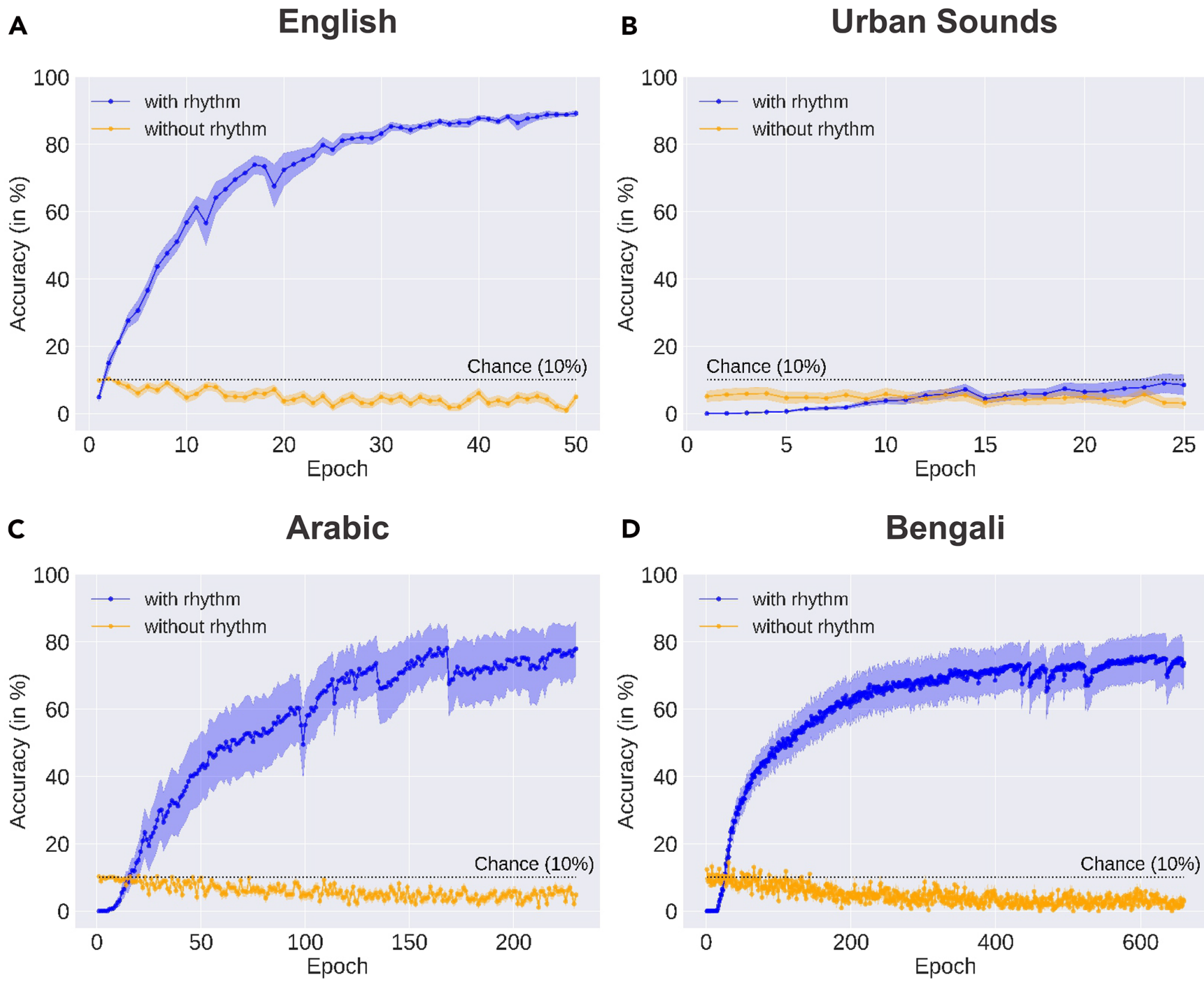

Figure 3. Performance with and without network oscillations

Test accuracy of networks configured with oscillation frequencies chosen uniformly at random from the range [0.1, 20] Hz (blue), compared to the same networks without oscillations (yellow). The accuracy after every training epoch is shown. Values are averaged across ten random seeds. The shaded region depicts the standard error. The datasets used were (A) AudioMNIST (English), (B) UrbanSound8k (Urban Sounds), (C) Arabic Spoken Digits, and (D) Bengali Spoken Digits.

(5% ± 2% for Arabic and 3% ± 1% for Bengali; Figures 3C and 3D). Networks configured with higher-frequency oscillations (>20 Hz) are unable to learn, with performance dropping to chance levels (Figures 4B and 4C), again showing that a match between signal and network oscillations is necessary for high task performance.

To examine which features of the spoken words are most critical for learning, we decompose the speech waveforms in the English dataset into mel-frequency cepstral coefficients (MFCCs), which extract frame-by-frame spectral content from continuous audio signals. The first MFCC computes the overall log energy of the signal and closely tracks the speech envelope (Figure S1B), while higher-order coefficients capture progressively finer spectral details (Figure S1C). When the first MFCC is provided as input to the high-performing oscillatory networks, they successfully learn the task (Figure S2B). However, this did not hold for any of the other coefficients. For example, when the same networks are given the seventh MFCC, performance remains at chance level (Figure S2C). This indicates that the oscillatory networks preferentially extract envelope-level information from the raw waveform. They achieve high performance by tracking the temporal modulations of the envelope, which is the most salient structure in the signal. Networks without endogenous oscillations, which are unable to learn the task using the raw waveform, achieve high performance when given the first MFCC, since the MFCC transform extracts envelope information by design (Figure S2B).

Because low-frequency oscillations in the 1–5 Hz range result in the best performance across all three languages, we next assess whether the representations learned for one language can be used to speed up training for another language. To test this, we take a network trained on spoken English digits and use it to initialize training on spoken Arabic and Bengali digits. We find that networks initialized from the English-trained model learn Arabic and Bengali faster (within only a few epochs) and to near-perfect accuracy, compared to the same networks trained from scratch (which took over 100s of epochs; Figures S3A and S3B). This improvement suggests that the English-trained network learns generic temporal regularities in speech, which provides a favorable starting point for learning new languages. The results of this transfer learning experiment are consistent with the observation that the speech envelope across all three languages peaks in the same 1–5 Hz range (Figure 1C).

Performance for non-speech sounds

Next, we examine whether the results for speech extend to non-speech stimuli using a dataset of urban sounds.[18] Urban sounds, such as traffic or construction noise, lack the distinct temporal modulations characteristic of speech. We test whether networks configured with low-frequency oscillations still provide a performance advantage when trained to classify sounds in this dataset. The results indicate that these networks are no more effective than non-oscillatory networks in this task. Performance values are 8% ± 3% for networks with oscillations and 2% ± 2% for networks without oscillations (Figure 3B). This provides further support for our hypothesis that endogenous oscillations serve as prior knowledge of signal structure—the priors that are highly effective at speech recognition are entirely ineffective in recognizing signals that lack speech-like structure.

Speech rhythms can vary naturally across speakers and contexts, prompting the question of how such variability affects models with fixed temporal assumptions. In particular, we asked whether artificially altering the speech rate—by slowing it down (0.5×) or speeding it up (2×)—impacts our highest-performing networks. The results show a significant drop in performance of ∼20% when we either speed up or slow down the speech rate, compared to the natural speech rate (Figure S4). Although the network retains some capacity to learn under altered speech rates, the performance drop shows the dependency of recognition on signal-network alignment. A natural way to incorporate full speech-rate invariance would be to allow the intrinsic frequencies to adapt online or during learning rather than remain fixed.[19]

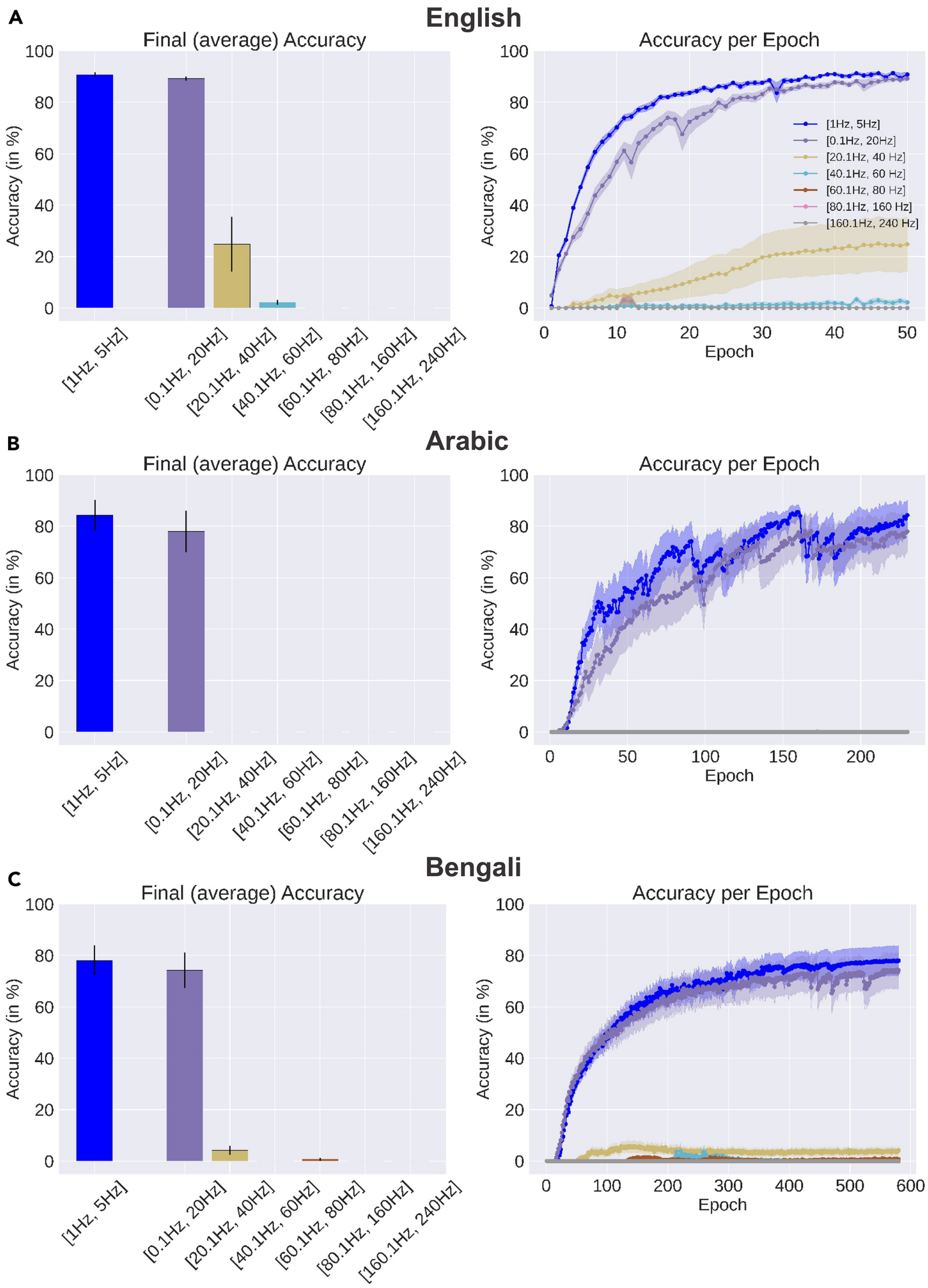

Figure 4. Performance across oscillation frequencies

(A) Accuracy in detecting English spoken words. Left: test accuracy of networks configured with oscillator frequencies drawn uniformly at random from the specified ranges. Values are averaged across ten random seeds. Error bars depict the standard error. Performance is highest for low-frequency oscillations that match the temporal structure of the signal. Right: test accuracy per epoch of the same networks.

(B and C) Arabic and Bengali.

To further examine signal and network alignment, we train networks on synthetic sinusoidal inputs of fixed frequencies. The network oscillators are initialized with frequencies drawn from fixed ranges, and their task is to classify the frequency of the input signal. This setup allows us to directly test whether matching signal and network rhythms facilitate task learning. When the task is to classify sinusoids drawn from the frequency range 10–100 Hz (requiring the signal to be classified into one of 10 uniformly spaced frequency bins), a network with oscillator frequencies drawn from the same range learns rapidly, achieving nearly 99% accuracy (Figure 5). In contrast, a network with oscillator frequencies drawn from the range 1–10 Hz performs at chance level, while a network with frequencies drawn from the range 100–110 Hz learns the task slowly, plateauing at around 80% accuracy. As a control, a network without intrinsic oscillations also learns the task slowly and plateaus at 80%. The non-oscillatory network has moderate performance in this task because the synthetic sinusoids were pure tones with no variability beyond amplitude and phase.

Importantly, oscillatory networks that achieve moderate or high performance in this sinusoid recognition task exhibit learned rhythmic activity whose period matches the period of the input signal, whereas networks performing at chance do not show this signal-network alignment (Figure S5). These results reaffirm that a match between signal and network rhythms is essential for achieving high task performance. They also indicate that while networks with low-frequency endogenous oscillations are unable to detect features of faster inputs, those with high frequency retain the ability to adapt downward to slower inputs in this particular task.

Effects of damping

Each node in our network is parameterized by a fixed frequency coefficient γ and a fixed damping coefficient ϵ (methods). The choices of these coefficients bias the learning process toward particular oscillations. In the results presented so far, we have examined the effects of the frequency coefficient while keeping the damping unchanged across the setups. In this section, we vary the damping coefficient and assess its effects on task performance.

First, in the spoken English words task, we set the frequency coefficient to zero and chose damping values for each unit uniformly at random from the range [0.1, 80]. We observe that this network initially struggles with the task but gradually reaches high performance with continued training (Figure S6). High performance is achieved once the network learns to oscillate at the frequency of the speech envelope (~1 Hz; Figure S7). In contrast to this slow learning, if the same network is initialized with frequency coefficients matched to the frequency of the speech envelope, it requires fewer training epochs to reach high performance, learning the task more rapidly (Figure S6).

Next, we perform a systematic scan across a wide range of damping coefficients and assess performance. For this,

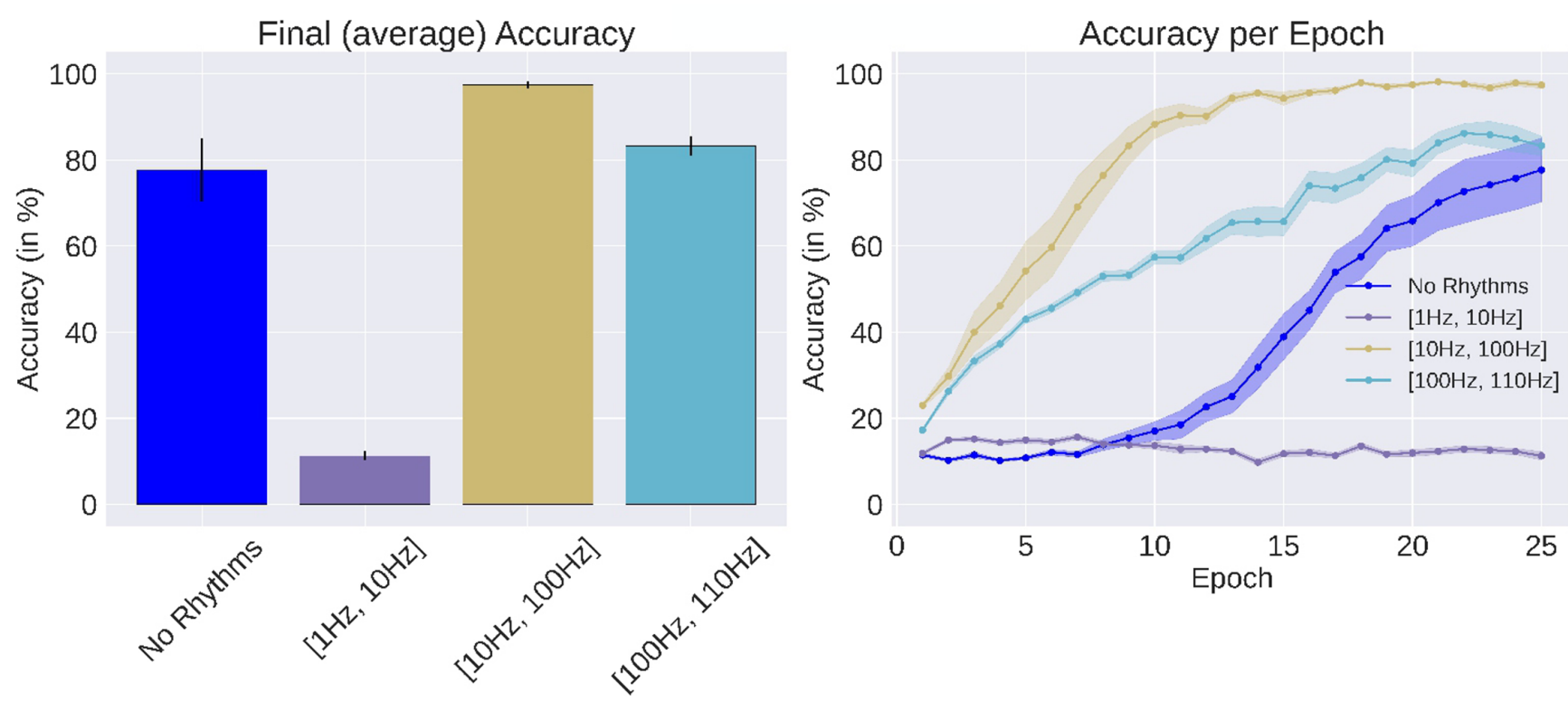

Figure 5. Performance in classifying synthetic sinusoidal inputs drawn from the range 10–100 Hz

Left: test accuracy of networks configured with oscillator frequencies drawn uniformly at random from the specified ranges. Values are averaged across ten random seeds. Error bars depict the standard error. Networks with matching intrinsic frequencies (10–100 Hz) achieve the best performance. Right: test accuracy per epoch of the same networks.

networks are initialized with frequencies that match the speech envelope, while the damping range is varied across intervals of a progressively increasing range. We find that when damping is too low (or absent), networks perform poorly (Figure S8A); for example, test accuracy is 5% when the damping is chosen uniformly at random from the range [0.1,5] for each unit. Performance improves once damping is increased (e.g., to the range [0.1,45]), but when damping becomes too large, performance drops again (Figure S9). These results indicate that there is a sweet spot for damping: too little damping prevents network oscillations from stabilizing to the task, while too much damping suppresses oscillations altogether (Figure S10).

State-space trajectories

To visualize the dynamics of the networks, we plot the first three principal components of network activity in response to spoken words, using network configurations that achieved the best task performance. The first three principal components capture 94%, 98%, and 91% of the total variance in networks trained on English, Arabic, and Bengali, respectively. We find that before training, network trajectories across words and languages are almost identical, with only minor variations reflecting subtleties in intonation and stress patterns (Figures 6A–6C, untrained). During training, the trajectories become increasingly distinct for each spoken word, and once training is complete, the trajectories are highly decorrelated, encoding the specific patterns of each word across the three languages (Figures 6A–6C, trained). In contrast, networks that lack intrinsic oscillations do not learn decorrelated representations across words and languages (Figure 7; the first three principal components capture 99% of the total variance in these networks for all three languages). The scaffold provided by the low-frequency oscillations, therefore, enables class-wise trajectory separation via training, which underlies high classification accuracy (Figure 3).

We then assess which oscillators predominantly shape activity when a network is initialized with frequencies drawn uniformly at random from the range [0.1, 20 Hz]. For this, we calculate the component loadings of the first principal component of the network's activity after training. The first principal component captures ~70% of the total variance. Across the three languages, we observe that oscillators within 0.1 and 2.5 Hz dominate the network's activity (Figure 8), with higher-frequency oscillators (>5 Hz) exerting only a weak influence. Thus, consistent with our other results, low-frequency oscillations are the primary drivers of computation in the network.

DISCUSSION

Summary

We show that networks of coupled oscillators with endogenous low-frequency oscillations accurately learn to recognize spoken words in three distinct languages. Our results demonstrate that oscillations serve as prior knowledge of speech structure, providing a temporal scaffold on which statistical learning takes place. Unit oscillations drawn from the 1–5 Hz frequency range result in the best task performance in all three languages. This suggests that despite differences in phonetic and prosodic features of English, Arabic, and Bengali, their raw acoustic signals share common, perhaps universal, temporal structure. It has been proposed that speech production involves quasi-rhythmic motor patterns dictated by skeletal, muscular, and neural-circuit-level constraints of the human speech-motor system.[5,20,21] The perceptual system, in turn, imposes rhythms that are tuned to detect and interpret speech signals at the appropriate temporal granularity, akin to active sensing in other modalities, but without explicit movement-driven sampling. Our results lend computational support to this view.

Signal and network oscillations

Our results indicate that imposing oscillatory dynamics in itself is insufficient for extracting task-relevant structure. Rather, a match between signal and network oscillations is essential. We find that networks with intrinsic oscillations that mirror those of the audio input reach high speech recognition accuracy, whereas networks with oscillations mismatched to the signal are unable to learn the task. In circuit theory, frequency matching between system and input, or resonance, imparts frequency selectivity, voltage magnification, and other signal transformations, widely used in analog communication systems. In analogy, circuit resonance in the brain has been reported in several studies, where signals arriving in phase with the natural oscillations of cortical circuits are selectively gated or amplified.[22–24] In our model, we use an explicit parameter γ to define the characteristic frequency of each node and suggest it be interpreted as a *dynamic* inductive bias that

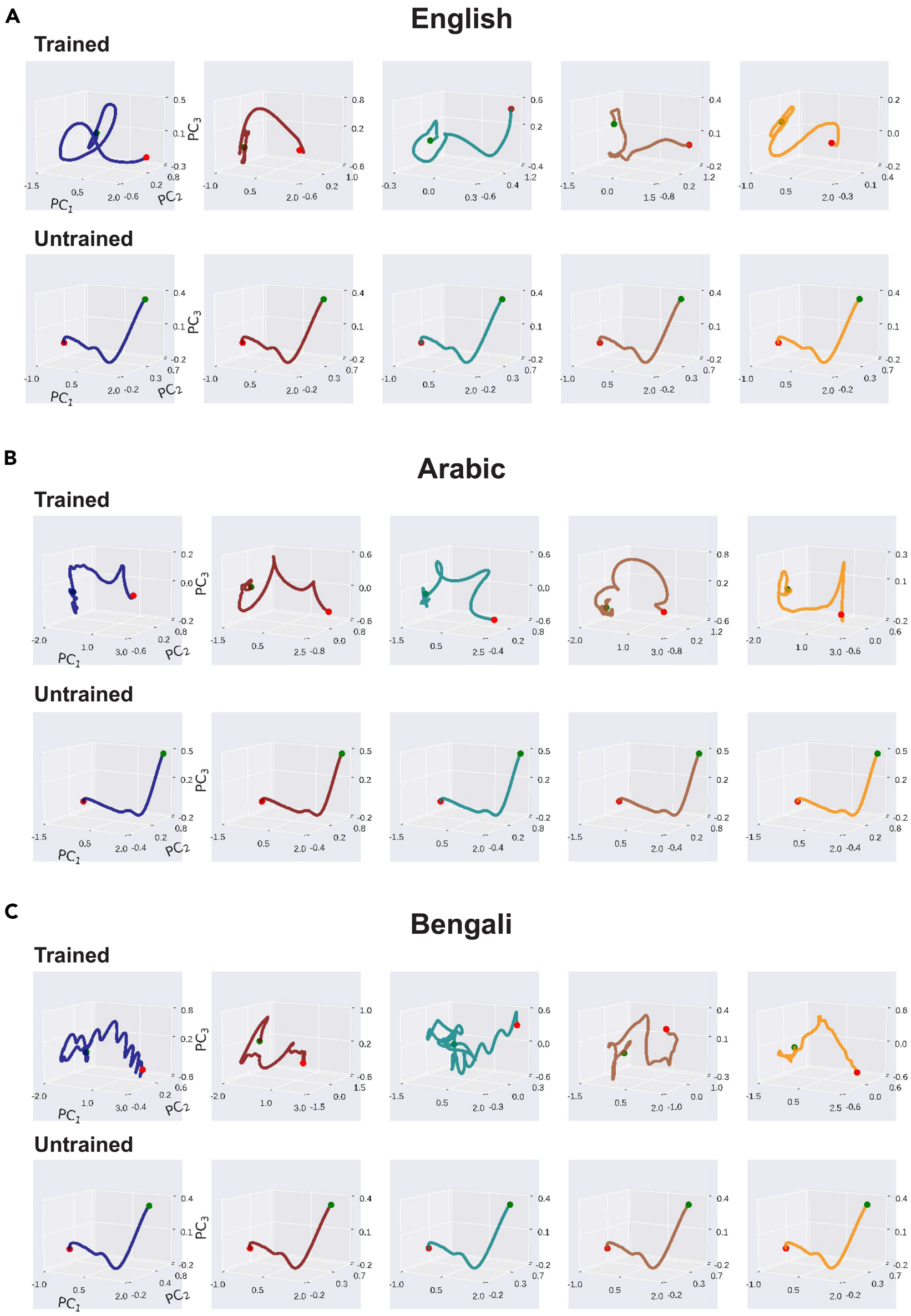

Figure 6. Network trajectories with endogenous oscillations

(A) Trajectory of network activity in principal-component analysis (PCA) space in response to five spoken digits in English ("zero," "two," "four," "six," and "eight"). Trajectories are averaged across all test samples in the dataset. The network is initialized with frequencies drawn uniformly at random from the range [0.1, 20] Hz. Green and red dots depict the start and end of the trajectories, respectively. Top: after training. Bottom: before training.

(B and C) Arabic and Bengali.

enables effective categorization of signals with matching temporal structure. We note that although the initial resonance properties of each unit are determined by γ, the learned frequency response is more complex due to network influences, as also reported in other studies.[12,13]

Interacting oscillations

While our model demonstrates that individual low-frequency oscillators matched to the speech envelope serve as effective inductive biases, the broader literature suggests that signal transformations in the brain likely emerge from coordinated interactions among multiple rhythms operating at different timescales rather than any single frequency band. Empirical results indicate that interactions among brain rhythms are organized into hierarchical networks with coupling patterns that differ systematically across distinct physiological states.[25,26] These patterns are characterized by specific functional forms—including stable positive correlations, stable anti-correlations, and state-dependent mixed interactions—and unique ensembles of these coupling forms serve as hallmarks of functional brain states. In the context of speech, such cross-frequency coupling could allow a network to simultaneously track slow envelope dynamics for segmentation while using faster oscillations to resolve finer phonetic detail within each segment.[14,27] These observations suggest that inductive biases for speech recognition may be rooted in multi-timescale, coordinated interactions among brain rhythms rather than in a single frequency band. This motivates future extensions of the coupled oscillatory RNN (coRNN) architecture in which coupling between oscillators at different timescales is explicitly constrained or initialized to reflect the hierarchical network interactions observed in the brain.

Inductive biases

In machine learning, inductive biases, or priors, refer to pre-existing knowledge or initial assumptions given to a model as cues to guide the learning process. This enables faster and more efficient training. A prior may be encoded by imposing a particular structure on the form of a model or as a hint about the underlying distribution of the input (e.g., sparsity regularization in LASSO). Whether explicit or implicit, priors lend direction to the learning algorithm to find meaningful patterns in the data, narrowing the range of possible hypotheses about the class of functions being learned.[28] In a neural network, finding good minima in a high-dimensional, non-convex optimization landscape can be difficult.[29] Priors help circumvent this difficulty by initializing the network to favorable configurations, enabling it to avoid the adverse terrain of the landscape.

In the brain, priors are encoded during development, though their precise forms are not well understood. They may take the form of stereotyped circuit architectures imposed by development[30–33] or, as we propose here, particular types of dynamical response. Learning involves adapting these initial expectations to the patterns of stimuli encountered in the world. For example, in language acquisition, priors may encode general syntactic

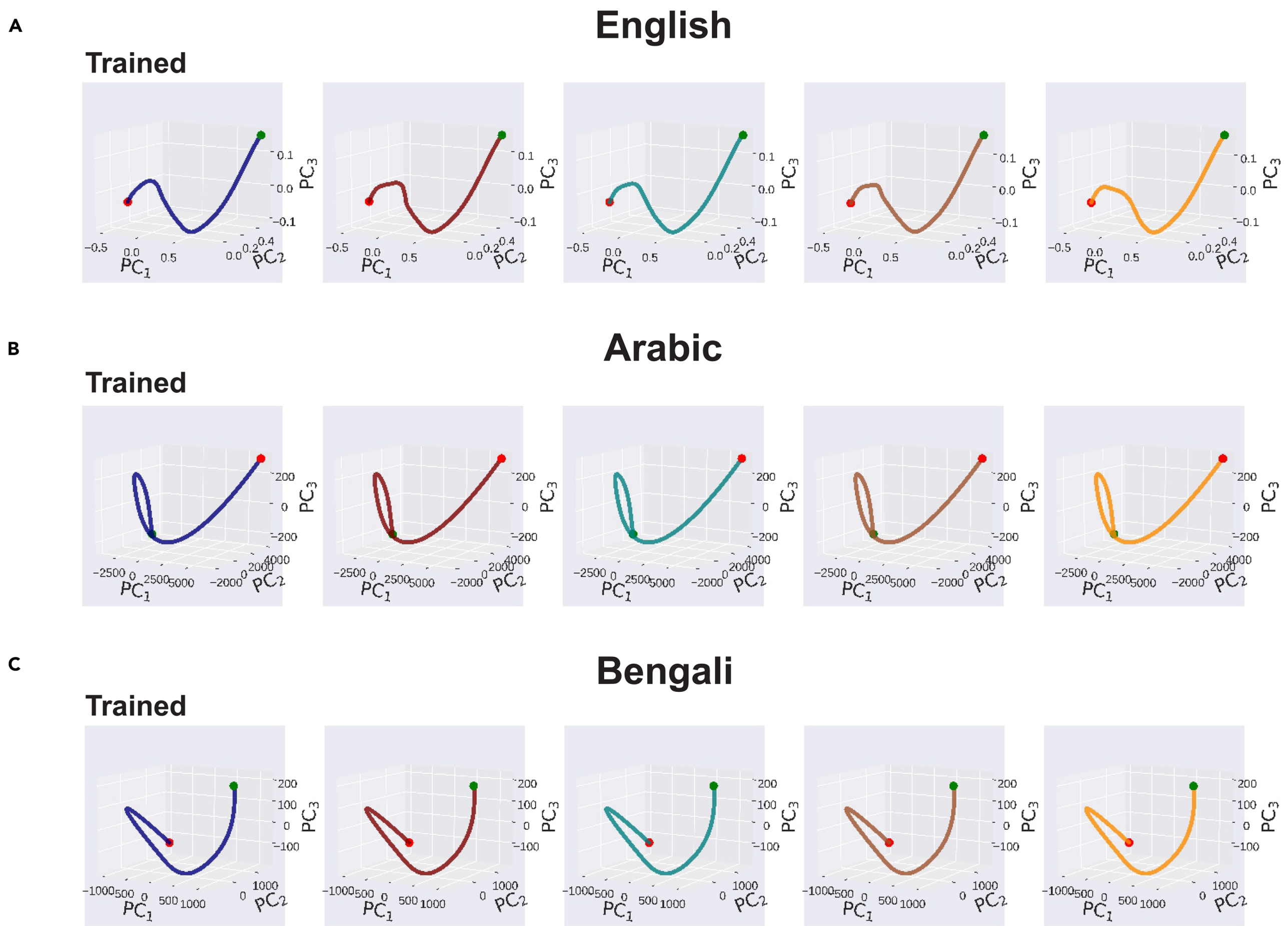

Figure 7. Network trajectories without endogenous oscillations

(A) Trajectory of network activity in PCA space in response to five spoken digits in English (“zero,” “two,” “four,” “six,” and “eight”) for a network with no endogenous oscillations and randomly initialized weights. Different digits evoke the same trajectory. Trajectories are averaged across all test samples in the dataset. Green and red dots depict the start and end of the trajectories, respectively.

(B and C) Arabic and Bengali.

constraints, with exposure to a particular language resulting in language-specific expectations and associations with sound. The learning rules in the brain likely differ substantially from the BPTT algorithm used here,[34–37] although alternative forms of credit assignment have been proposed.[38–40]

Engineered systems

The results presented here are not comparisons against state-of-the-art automatic speech recognition systems,[41,42] which achieve high performance in a variety of tasks using large-scale models and vast amounts of data. Whereas these systems learn speech representations through self-supervised pretraining with scalable network architectures, the networks presented here use coupled oscillators with no feature extraction or pretraining and are designed to test a functional hypothesis about brain oscillations. However, recent speech synthesis systems such as Qwen3-TTS[43] quantize speech representations at frequencies of 12.5 and 25 Hz—producing high-fidelity reconstructions of speech signals—suggesting that engineered systems can benefit from exploiting the low-frequency temporal structure of speech signals for speech recognition and generation tasks.

Wave-RNNs

In addition to the standing-wave models presented here, neural systems exhibit other classes of rich temporal structure. For example, traveling waves of activity patterns that unfold across the cortical surface are a topic of active investigation.[44] When embedded within RNNs, traveling waves generate dynamic representations that can be exploited for efficient sequence learning, continual learning, and long-term memory consolidation.[45–49] Other phenomena characteristic of coupled neural systems, including transient synchronization, phase locking, and the hierarchical nesting of oscillations, may also be incorporated into similar wave-based models.[9,10,50–54] Among these, synchronization is perhaps the most well studied, with the Kuramoto model providing a canonical example illustrating how populations of coupled oscillators spontaneously synchronize through phase interactions.[55] In this model, each oscillator is driven by its own intrinsic frequency but is pulled toward the phases of its

CellPress
OPEN ACCESS

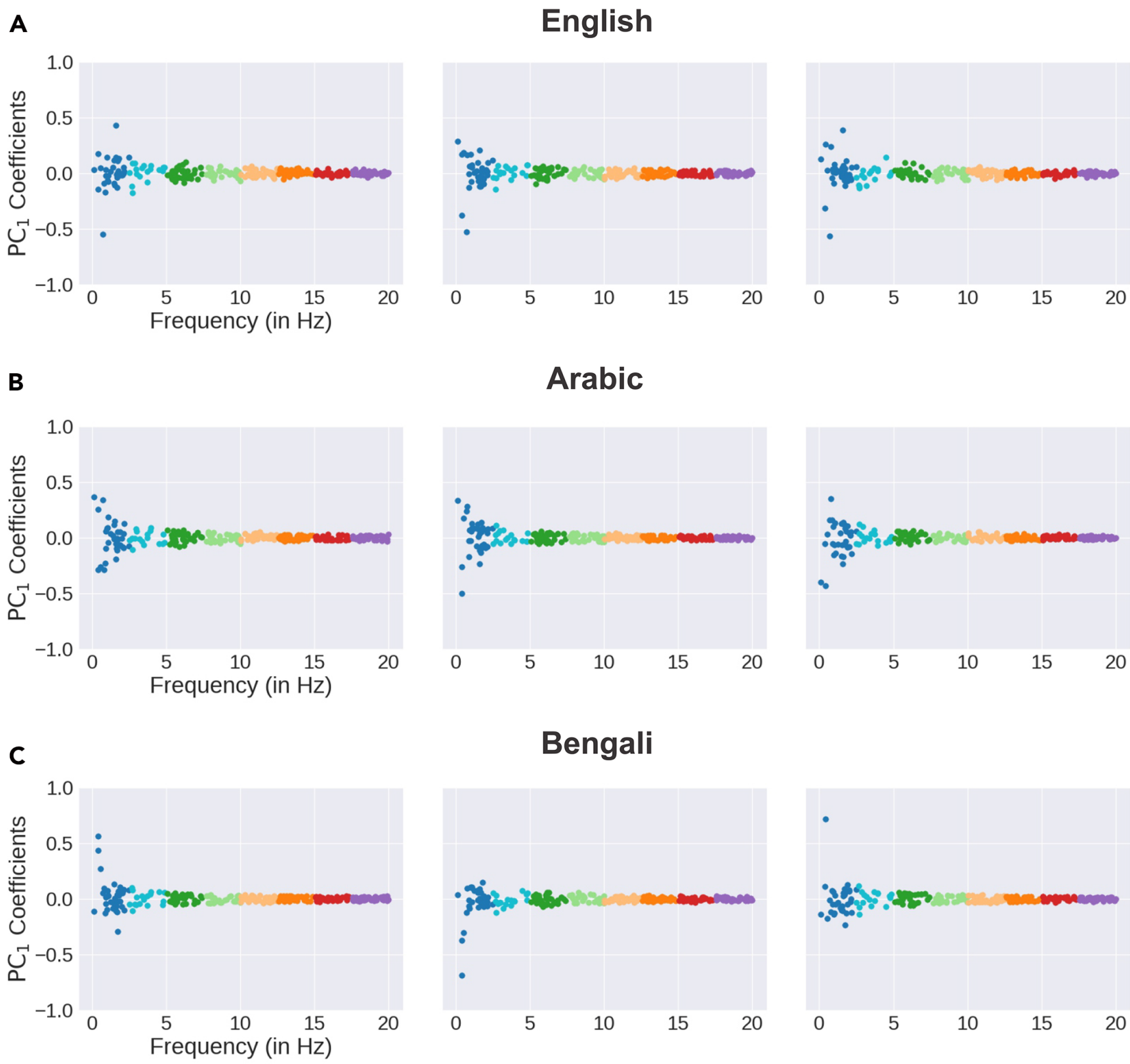

Figure 8. Dominant oscillation mode
The influence of each oscillator on the first principal component in response to three different spoken words (three separate columns) for (A) English, (B) Arabic, and (C) Bengali for a network trained with randomly initialized frequencies from the range [0.1, 20] Hz.

neighbors through sinusoidal coupling. When the coupling strength exceeds a critical threshold, the population locks into a collective rhythm. This dynamic can be embedded in neural network layers in place of traditional threshold-based activation functions to exploit synchronization as a mechanism for feature binding and representation learning.[56] These varieties of wave-based RNNs offer new tools to artificial intelligence and serve as platforms for testing computational hypotheses about neural circuit function.

METHODS

Datasets

We used the AudioMNIST (English), Arabic Spoken Digits, BanglaNum (Bengali), and UrbanSound8K datasets in our tasks. AudioMNIST consists of 30,000 audio recordings ($\sim$8.3 h) of spoken digits (0 through 9) across 60 different English speakers.[57] Each recording is $\sim$1 s, originally sampled at 48 kHz on a single channel. Each speaker recited each digit 50 times, resulting in 3,000 recordings across each class.

The Arabic Spoken Digits dataset is a subset of the Arabic Speech Corpus for Isolated Words.[58] The dataset consists of approximately 5,000 audio recordings of spoken digits (0 through 9) across 20 different Arabic speakers. Each recording is $\sim$1 s, originally sampled at 44,100 Hz on two channels (i.e., stereo). The Bengali Spoken Digits (BanglaNum) dataset consists of approximately 2,250 audio recordings of spoken digits (0 through 9) across 40 different Bengali speakers.[59] Each recording is $\sim$0.5 s, originally sampled at 16 kHz using a standard single-channel microphone.

UrbanSound8K consists of field recordings of various sounds found in an urban environment, spanning 10 low-level classes (i.e., air conditioner, car horn, children playing, dog bark, drilling, engine idling, gunshot, jackhammer, siren, and street music).[18] In total, there are 8,732 audio recordings ($\sim$7.06 h), each with a variable time length ($\leq$4 s) with sampling rates varying from 8 to 96 kHz.

For the language datasets, we randomly select 90% of the samples to be used as the training set and the rest as the test set. For urban sounds, we randomly select 80% as the training set and the rest as the test set. For all datasets, we first downsample each recording to 8 kHz and then normalize the amplitude of the signal to the interval $[-1,1]$. The resulting time series, $I(t)$, is used as the input signal to the network.

For the mel-frequency experiments, the raw waveform was transformed into MFCCs before training. Each signal was processed with an MFCC transform that extracted 13 coefficients per frame, using a 200-point short-time Fourier transform (STFT) with a hop length of 5 samples and a 20-channel mel filterbank. The resulting time-frequency representation was used as the network input.

For the sinusoidal experiments, we generated synthetic waveforms of fixed frequencies. The waveform was defined as $s(t) = A\sin(2\pi ft + \phi)$, where A is the amplitude, f is the frequency, t is time, and ϕ is the phase shift. For each frequency, we created multiple examples by sampling the amplitude uniformly from [0.1,5] and the phase shift uniformly from $[0,2\pi)$. Using this procedure, we generated 10 classes of sinusoids corresponding to fixed frequencies of 10–100 Hz, spaced equally. An analogous dataset was also generated for the low-frequency range [1,10] Hz, with classes defined by fixed frequencies of 1–10 Hz. Each class consisted of 1,000 samples. We randomly selected 80% of the samples to be used as the training set and the rest as the test set. These waveforms then served as the input to the network.

Model description

We use the coRNN setup, an RNN architecture of time-discretized coupled oscillators.[13] In the coRNN, the coupled oscillators take the form of the following second-order, m-dimensional system of ODEs:

$$\mathbf{y}''(t) = \sigma\left(W^{Rec}\mathbf{y}(t) + W^{Vel}\mathbf{y}'(t) + W^{in}\mathbf{I}(t) + \mathbf{b}\right) - \gamma\mathbf{y}(t) - \epsilon\mathbf{y}'(t), \quad \text{(Equation 1)}$$

where $\mathbf{I}(t) \in \mathbb{R}^d$ is the time-varying input signal; $W^{in} \in \mathbb{R}^{m\times d}$ is the input weight matrix; $\mathbf{y}(t) \in \mathbb{R}^m$ is the hidden states of the RNN; $\mathbf{y}'(t) \in \mathbb{R}^m$ is the velocity of hidden states of the RNN; $W^{Rec}, W^{Vel} \in \mathbb{R}^{m\times m}$ is the recurrent weight matrices; $\mathbf{b} \in \mathbb{R}^m$ is the bias term; $\sigma(\cdot) \in \mathbb{R}$ is the activation function (tanh); $\boldsymbol{\gamma} \in \mathbb{R}^m$ is the characteristic frequency (Hz) of each node ($\geq$0); and $\epsilon \in \mathbb{R}^m$ is the damping of each node ($\geq$0)

Each node in the network is parameterized by a fixed characteristic frequency (γ) and damping (ϵ) coefficient. Time, $t \in [0,1]$, represents a continuous variable. To simulate Equation 1, we let the velocity term $\mathbf{y}'(t) = \mathbf{z}(t)$. We can then rewrite the second-order ODE (Equation 1) as a first-order ODE (Equation 2), where $\mathbf{z}'(t) = \mathbf{y}''(t)$, resulting in

$$\mathbf{z}'(t) = \sigma\left(W^{Rec}\mathbf{y}(t) + W^{Vel}\mathbf{z}(t) + W^{in}\mathbf{I}(t) + \mathbf{b}\right) - \gamma\mathbf{y}(t) - \epsilon\mathbf{z}(t). \quad \text{(Equation 2)}$$

For a fixed timestep, $0 < \Delta t < 1$, the IMEX (implicit-explicit) discretization of this equation results in

$$\mathbf{y}_t = \mathbf{y}_{t-1} + \mathbf{z}_t\Delta t \text{ and} \quad \text{(Equation 3)}$$

$$\mathbf{z}_t = \mathbf{z}_{t-1} + \Delta t\sigma\left(W^{Rec}\mathbf{y}_{t-1} + W^{Vel}\mathbf{z}_{t-1} + W^{in}I_t + \mathbf{b}\right) - \Delta t\gamma\mathbf{y}_{t-1} - \Delta t\epsilon\mathbf{z}_{t-1}. \quad \text{(Equation 4)}$$

We consider only the explicit discretization of Equations 3 and 4 in the network simulations.

We simulate the coRNN both with and without endogenous frequencies, using a network of $m = 256$ units in each case. In the endogenous case, we configure the network with various combinations of oscillations and damping (Table S1). To configure networks without endogenous oscillations, we set γ and ϵ to 0 in Equation 1, effectively resulting in a standard (Elman) RNN.[60] All networks have an equivalent number of trainable parameters. The all-to-all recurrent coupling matrix W^{rec} enables interactions among oscillators operating at different frequencies.

Network training

All simulated networks consist of a trainable input, recurrent, and readout (SoftMax) layer, where the initial weights are sampled from a uniform distribution, $W \sim \mathcal{U}\left(-\frac{1}{\sqrt{n}}, \frac{1}{\sqrt{n}}\right)$ (n is the input dimension to each layer). The input to each network, $I(t)$, is a time-varying signal. All parameters were subsequently trained via BPTT. We used the cross-entropy loss function with the AdamW optimizer, a learning rate of 4.2×10^{-3}, and a time step of 5×10^{-3}.

For the AudioMNIST, Arabic Spoken Digits, and Bengali Spoken Digits datasets, we used 50 training epochs with a batch size of 64, which took a total of ~50 h of training time per network on an NVIDIA V100 GPU. For the UrbanSounds8k dataset, we used 25 epochs with a batch size of 32, which took ~25 h to train. We carried out a systematic scan of the frequency and damping ranges (Table S1) but did not perform an exhaustive grid search due to the long training times.

Network readout

To assess network performance on the speech tasks, we used the following classification scheme: for the last ~62.5 ms of the input signal, we take the node in the SoftMax layer with the highest activation. Specifically, if the maximum SoftMax activation at time t exceeds a threshold of 0.60, the corresponding class i is assigned as the prediction at that time point; otherwise, the output is labeled as null. After collecting predictions across the classification window, we determined the final class label as follows: if class i is the most frequently predicted label in this window and appears in at least 80% of the non-null predictions, then i is selected as the network's final decision. Otherwise, the classification is marked as uncertain. This scheme ensures robust classification at readout despite the oscillatory nature of the network. See Figure 2C for an illustration of a network response.

For the sinusoidal classification task, we adopted a complementary readout strategy appropriate for fixed-frequency sinusoids. Rather than using only the final segment of the input, we concatenated the hidden-state activity of all network units across the entire temporal window into a single vector for each trial. A linear readout layer (SoftMax classifier) was then trained on this aggregated representation to predict the frequency class. This approach gave the classifier access to the entire trajectory of network dynamics, allowing it to capture the oscillatory patterns that persisted throughout the input.

RESOURCE AVAILABILITY

Lead contact

Requests for further information and resources should be directed to and will be fulfilled by the lead contact, Nabil Imam (nimam6@gatech.edu).

Materials availability

This study did not generate new, unique reagents.

Data and code availability

The code to reproduce the results of this study is available as a GitHub repository: https://github.com/nandchandravadia/rhythmRNNs_speech. The code is also available at a Zenodo repository: https://doi.org/10.5281/zenodo.19411357.[61]

ACKNOWLEDGMENTS

This work was supported by NSF grants 2319060 and 2223811.

AUTHOR CONTRIBUTIONS

Conceptualization, N.C. and N.I.; methodology, N.C. and N.I.; data curation, N.C.; software, N.C.; validation, N.C.; formal analysis, N.C. and N.I.; investigation, N.C. and N.I.; writing, N.C. and N.I.; visualization, N.C. and N.I.; supervision, N.I.

DECLARATION OF INTERESTS

The authors declare no competing interests.

SUPPLEMENTAL INFORMATION

Supplemental information can be found online at https://doi.org/10.1016/j.patter.2026.101563.

CellPress
OPEN ACCESS

Received: November 1, 2025
Revised: March 3, 2026
Accepted: April 21, 2026
Published: May 19, 2026

REFERENCES

1. Giraud, A.L., Kleinschmidt, A., Poeppel, D., Lund, T.E., Frackowiak, R.S.J., and Laufs, H. (2007). Endogenous cortical rhythms determine cerebral specialization for speech perception and production. Neuron *56*, 1127–1134. https://doi.org/10.1016/j.neuron.2007.09.038.
2. Schroeder, C.E., and Lakatos, P. (2009). Low-frequency neuronal oscillations as instruments of sensory selection. Trends Neurosci. *32*, 9–18. https://doi.org/10.1016/j.tins.2008.09.012.
3. Luo, H., Liu, Z., and Poeppel, D. (2010). Auditory cortex tracks both auditory and visual stimulus dynamics using low-frequency neuronal phase modulation. PLoS Biol. *8*, e1000445. https://doi.org/10.1371/journal.pbio.1000445.
4. Ding, N., Melloni, L., Zhang, H., Tian, X., and Poeppel, D. (2016). Cortical tracking of hierarchical linguistic structures in connected speech. Nat. Neurosci. *19*, 158–164. https://doi.org/10.1038/nn.4186.
5. Poeppel, D., and Assaneo, M.F. (2020). Speech rhythms and their neural foundations. Nat. Rev. Neurosci. *21*, 322–334. https://doi.org/10.1038/s41583-020-0304-4.
6. Rosen, S. (1992). Temporal information in speech: acoustic, auditory and linguistic aspects. Philos. Trans. R. Soc. Lond. B Biol. Sci. *336*, 367–373. https://doi.org/10.1098/rstb.1992.0070.
7. Ding, N., Patel, A.D., Chen, L., Butler, H., Luo, C., and Poeppel, D. (2017). Temporal modulations in speech and music. Neurosci. Biobehav. Rev. *81*, 181–187. https://doi.org/10.1016/j.neubiorev.2017.02.011.
8. Ghitza, O., and Greenberg, S. (2009). On the possible role of brain rhythms in speech perception: intelligibility of time-compressed speech with periodic and aperiodic insertions of silence. Phonetica *66*, 113–126. https://doi.org/10.1159/000208934.
9. Buzsáki, G., and Vöröslakos, M. (2023). Brain rhythms have come of age. Neuron *111*, 922–926. https://doi.org/10.1016/j.neuron.2023.03.018.
10. Hoppensteadt, F.C., and Izhikevich, E.M. (2012). Weakly Connected Neural Networks, *126* (Springer Science & Business Media). https://doi.org/10.1007/978-1-4612-1828-9.
11. Wilson, H.R., and Cowan, J.D. (1972). Excitatory and inhibitory interactions in localized populations of model neurons. Biophys. J. *12*, 1–24. https://doi.org/10.1016/S0006-3495(72)86068-5.
12. Effenberger, F., Carvalho, P., Dubinin, I., and Singer, W. (2025). The functional role of oscillatory dynamics in neocortical circuits: a computational perspective. Proc. Natl. Acad. Sci. USA *122*, e2412830122. https://doi.org/10.1073/pnas.2412830122.
13. Rusch, T.K., and Mishra, S. (2021). Coupled oscillatory recurrent neural network (coRNN): An accurate and (gradient) stable architecture for learning long time dependencies. In International Conference on Learning Representations (ICLR). https://doi.org/10.48550/arXiv.2010.00951.
14. Giraud, A.L., and Poeppel, D. (2012). Cortical oscillations and speech processing: emerging computational principles and operations. Nat. Neurosci. *15*, 511–517. https://doi.org/10.1038/nn.3063.
15. Ghitza, O. (2011). Linking speech perception and neurophysiology: speech decoding guided by cascaded oscillators locked to the input rhythm. Front. Psychol. *2*, 130. https://doi.org/10.3389/fpsyg.2011.00130.
16. Werbos, P.J. (1990). Backpropagation through time: what it does and how to do it. Proc. IEEE *78*, 1550–1560. https://doi.org/10.1109/5.58337.
17. Goodfellow, I., Bengio, Y., Courville, A., and Bengio, Y. (2016). Deep Learning, *1* (MIT Press). http://www.deeplearningbook.org.
18. Salamon, J., Jacoby, C., and Bello, J.P. (2014). A Dataset and Taxonomy for Urban Sound Research (ACM Multimedia), pp. 1041–1044. https://doi.org/10.1145/2647868.2655045.
19. Dogonasheva, O., Giraud, A.L., Zakharov, D., and Gutkin, B. (2026). Neuro-oscillatory models of cortical speech processing. Neural Netw. *195*, 108194. https://doi.org/10.1016/j.neunet.2025.108194.
20. Guenther, F.H. (2016). Neural Control of Speech (MIT Press). https://doi.org/10.7551/mitpress/10471.001.0001.
21. MacNeilage, P.F. (1998). The frame/content theory of evolution of speech production. Behav. Brain Sci. *21*, 499–546. https://doi.org/10.1017/S0140525X98001265.
22. Knight, B.W. (1972). The relationship between the firing rate of a single neuron and the level of activity in a population of neurons: Experimental evidence for resonant enhancement in the population response. J. Gen. Physiol. *59*, 767–778. https://doi.org/10.1085/jgp.59.6.767.
23. Izhikevich, E.M. (2001). Resonate-and-fire neurons. Neural Netw. *14*, 883–894. https://doi.org/10.1016/S0893-6080(01)00078-8.
24. Hutcheon, B., and Yarom, Y. (2000). Resonance, oscillation and the intrinsic frequency preferences of neurons. Trends Neurosci. *23*, 216–222. https://doi.org/10.1016/S0166-2236(00)01547-2.
25. Lin, A., Liu, K.K.L., Bartsch, R.P., and Ivanov, P.C. (2020). Dynamic network interactions among distinct brain rhythms as a hallmark of physiologic state and function. Commun. Biol. *3*, 197. https://doi.org/10.1038/s42003-020-0878-4.
26. Chen, B., Ciria, L.F., Hu, C., and Ivanov, P.C. (2022). Ensemble of coupling forms and networks among brain rhythms as function of states and cognition. Commun. Biol. *5*, 82. https://doi.org/10.1038/s42003-022-03017-4.
27. Doelling, K.B., Arnal, L.H., and Assaneo, M.F. (2023). Adaptive oscillators support Bayesian prediction in temporal processing. PLoS Comput. Biol. *19*, e1011669. https://doi.org/10.1371/journal.pcbi.1011669.
28. Shalev-Shwartz, S., and Ben-David, S. (2014). Understanding Machine Learning: From Theory to Algorithms (Cambridge University Press). https://doi.org/10.1017/CBO9781107298019.
29. Li, H., Xu, Z., Taylor, G., Studer, C., and Goldstein, T. (2018). Visualizing the loss landscape of neural nets. In Advances in Neural Information Processing Systems, *31*. https://doi.org/10.48550/arXiv.1712.09913.
30. Imam, N., Kielo, M., Trude, B.M., and Finlay, B.L. (2026). Dual computational systems in the development and evolution of mammalian brains. Sci. Adv. *12*, eaec6112. https://doi.org/10.1126/sciadv.aec6112.
31. Imam, N., and L. Finlay, B. (2020). Self-organization of cortical areas in the development and evolution of neocortex. Proc. Natl. Acad. Sci. USA *117*, 29212–29220. https://doi.org/10.1073/pnas.2011724117.
32. Zador, A.M. (2019). A critique of pure learning and what artificial neural networks can learn from animal brains. Nat. Commun. *10*, 3770. https://doi.org/10.1038/s41467-019-11786-6.
33. Shuvaev, S., Lachi, D., Koulakov, A., and Zador, A. (2024). Encoding innate ability through a genomic bottleneck. Proc. Natl. Acad. Sci. USA *121*, e2409160121. https://doi.org/10.1073/pnas.2409160121.
34. Feldman, D.E. (2012). The spike-timing dependence of plasticity. Neuron *75*, 556–571. https://doi.org/10.1016/j.neuron.2012.08.001.
35. Pehlevan, C., Sengupta, A.M., and Chklovskii, D.B. (2018). Why do similarity matching objectives lead to hebbian/anti-hebbian networks? Neural Comput. *30*, 84–124. https://doi.org/10.1162/neco_a_01018.
36. Linster, C., and Cleland, T.A. (2010). Decorrelation of odor representations via spike timing-dependent plasticity. Front. Comput. Neurosci. *4*, 157. https://doi.org/10.3389/fncom.2010.00157.
37. Moore, J.J., Genkin, A., Tournoy, M., Pughe-Sanford, J.L., de Ruyter van Steveninck, R.R., and Chklovskii, D.B. (2024). The neuron as a direct data-driven controller. Proc. Natl. Acad. Sci. USA *121*, e2311893121. https://doi.org/10.1073/pnas.2311893121.
38. Gütig, R. (2016). Spiking neurons can discover predictive features by aggregate-label learning. Science *351*, aab4113. https://doi.org/10.1126/science.aab4113.
39. Bellec, G., Scherr, F., Subramoney, A., Hajek, E., Salaj, D., Legenstein, R., and Maass, W. (2020). A solution to the learning dilemma for recurrent networks of spiking neurons. Nat. Commun. *11*, 3625. https://doi.org/10.1038/s41467-020-17236-y.

40. Liu, Y.H., Smith, S., Mihalas, S., Shea-Brown, E., and Sümbül, U. (2021). Cell-type–specific neuromodulation guides synaptic credit assignment in a spiking neural network. Proc. Natl. Acad. Sci. USA *118*, e2111821118. https://doi.org/10.1073/pnas.2111821118.

41. Schneider, S., Baevski, A., Collobert, R., and Auli, M. (2019). wav2vec: Unsupervised pre-training for speech recognition. In Interspeech 2019, pp. 3465–3469. https://doi.org/10.48550/arxiv.1904.05862.

42. Radford, A., Kim, J.W., Xu, T., Brockman, G., McLeavey, C., and Sutskever, I. (2023). Robust speech recognition via large-scale weak supervision. In International Conference on Machine Learning (ICML), pp. 28492–28518. https://doi.org/10.48550/arXiv.2212.04356.

43. Hu, H., Zhu, X., He, T., Guo, D., Zhang, B., Wang, X., Guo, Z., Jiang, Z., Hao, H., Guo, Z., et al. (2026). Qwen3-tts technical report. Preprint at arXiv. https://doi.org/10.48550/arXiv.2601.15621.

44. Muller, L., Chavane, F., Reynolds, J., and Sejnowski, T.J. (2018). Cortical travelling waves: mechanisms and computational principles. Nat. Rev. Neurosci. *19*, 255–268. https://doi.org/10.1038/nrn.2018.20.

45. Keller, T.A., and Welling, M. (2023). Neural wave machines: learning spatiotemporally structured representations with locally coupled oscillatory recurrent neural networks. In International Conference on Machine Learning (ICML) https://proceedings.mlr.press/v202/keller23a.html.

46. Lanthaler, S., Rusch, T.K., and Mishra, S. (2023). Neural oscillators are universal. Adv. Neural Inf. Process. Syst. *36*, 46786–46806. https://doi.org/10.48550/arXiv.2305.08753.

47. Muller, L., Churchland, P.S., and Sejnowski, T.J. (2024). Transformers and cortical waves: encoders for pulling in context across time. Trends Neurosci. *47*, 788–802. https://doi.org/10.1016/j.tins.2024.08.006.

48. Keller, T.A., Muller, L., Sejnowski, T., and Welling, M. (2024). Traveling waves encode the recent past and enhance sequence learning. In International Conference on Learning Representations (ICLR). https://doi.org/10.48550/arXiv.2309.08045.

49. Tadros, T., Krishnan, G.P., Ramyaa, R., and Bazhenov, M. (2022). Sleep-like unsupervised replay reduces catastrophic forgetting in artificial neural networks. Nat. Commun. *13*, 7742. https://doi.org/10.1038/s41467-022-34938-7.

50. Buzsáki, G. (2010). Neural syntax: cell assemblies, synapsembles, and readers. Neuron *68*, 362–385. https://doi.org/10.1016/j.neuron.2010.09.023.

51. Brody, C.D., and Hopfield, J.J. (2003). Simple networks for spike-timing-based computation, with application to olfactory processing. Neuron *37*, 843–852. https://doi.org/10.1016/s0896-6273(03)00120-x.

52. Imam, N., and Cleland, T.A. (2020). Rapid online learning and robust recall in a neuromorphic olfactory circuit. Nat. Mach. Intell. *2*, 181–191. https://doi.org/10.1038/s42256-020-0159-4.

53. Yan, Y., Yang, Q., Wu, Y., Liu, H., Zhang, M., Li, H., Tan, K.C., and Wu, J. (2025). Efficient and robust temporal processing with neural oscillations modulated spiking neural networks. Nat. Commun. *16*, 8651. https://doi.org/10.1038/s41467-025-63771-x.

54. Bazhenov, M., Stopfer, M., Rabinovich, M., Huerta, R., Abarbanel, H.D., Sejnowski, T.J., and Laurent, G. (2001). Model of transient oscillatory synchronization in the locust antennal lobe. Neuron *30*, 553–567. https://doi.org/10.1016/s0896-6273(01)00284-7.

55. Kuramoto, Y. (1984). Chemical Oscillations, Waves, and Turbulence. In Springer Series in Synergetics (Springer-Verlag). https://doi.org/10.1007/978-3-642-69689-3.

56. Miyato, T., Löwe, S., Geiger, A., and Welling, M. (2025). Artificial Kuramoto oscillatory neurons. In International Conference on Learning Representations (ICLR). https://doi.org/10.48550/arXiv.2410.13821.

57. Becker, S., Vielhaben, J., Ackermann, M., Müller, K.R., Lapuschkin, S., and Samek, W. (2024). Audiomnist: Exploring explainable artificial intelligence for audio analysis on a simple benchmark. J. Franklin Inst. *361*, 418–428. https://doi.org/10.1016/j.jfranklin.2023.11.038.

58. Alalshekmubarak, A., and Smith, L.S. (2014). On improving the classification capability of reservoir computing for arabic speech recognition. In International Conference on Artificial Neural Networks, pp. 225–232. https://doi.org/10.1007/978-3-319-11179-7_29.

59. Mohammad, M.S., Zahid, A., and Iqbal, M.A. (2024). Banglanum–a public dataset for bengali digit recognition from speech. Preprint at arXiv. https://doi.org/10.48550/arXiv.2403.13465.

60. Elman, J.L. (1990). Finding structure in time. Cogn. Sci. *14*, 179–211. https://doi.org/10.1207/s15516709cog1402_1.

61. Chandravadia, N. (2025). Code for “Neural rhythms as priors of speech computations.” Zenodo. https://doi.org/10.5281/zenodo.19411357.

Patterns, Volume *7*

Supplemental information

Neural rhythms as priors of speech computations

Nand Chandravadia and Nabil Imam

Supplementary Information

Frequency Range	Damping Range	English	Arabic	Bengali
[1, 5] Hz	[0.1, 80]	**90.79% ± 0.84%**	**84.28% ± 6.08%**	**78.01% ± 5.82%**
[0.1, 20] Hz	[0.1, 80]	89.20% ± 0.88%	77.95% ± 8.05%	74.20% ± 6.89%
[20.1, 40] Hz	[20.1, 40]	24.81% ± 10.60%	0.00% ± 0.00%	4.15% ± 1.77%
[40.1, 60] Hz	[40.1, 60]	2.17% ± 0.90%	0.00% ± 0.00%	0.00% ± 0.00%
[60.1, 80] Hz	[60.1, 80]	0.00% ± 0.00%	0.00% ± 0.00%	0.73% ± 0.60%
[80.1, 160] Hz	[80.1, 160]	0.00% ± 0.00%	0.00% ± 0.00%	0.00% ± 0.00%
[160.1, 240] Hz	[160.1, 240]	0.00% ± 0.00%	0.00% ± 0.00%	0.00% ± 0.00%
[250.1, 330] Hz	[250.1, 330]	0.00% ± 0.00%	0.00% ± 0.00%	0.00% ± 0.00%
[400.1, 480] Hz	[400.1, 480]	0.00% ± 0.00%	0.00% ± 0.00%	0.00% ± 0.00%
[1000.1, 1080] Hz	[1000.1, 1080]	0.00% ± 0.00%	0.00% ± 0.00%	0.00% ± 0.00%
No Rhythm	No Damping	4.90% ± 1.55%	4.80% ± 1.52%	3.01% ± 1.45%

Table S1: Final test accuracy of networks configured with frequency and damping coefficients selected uniformly at random from the specified ranges. The bold values indicate the highest accuracies. Values are averaged (mean ± standard error) across 10 random seeds.

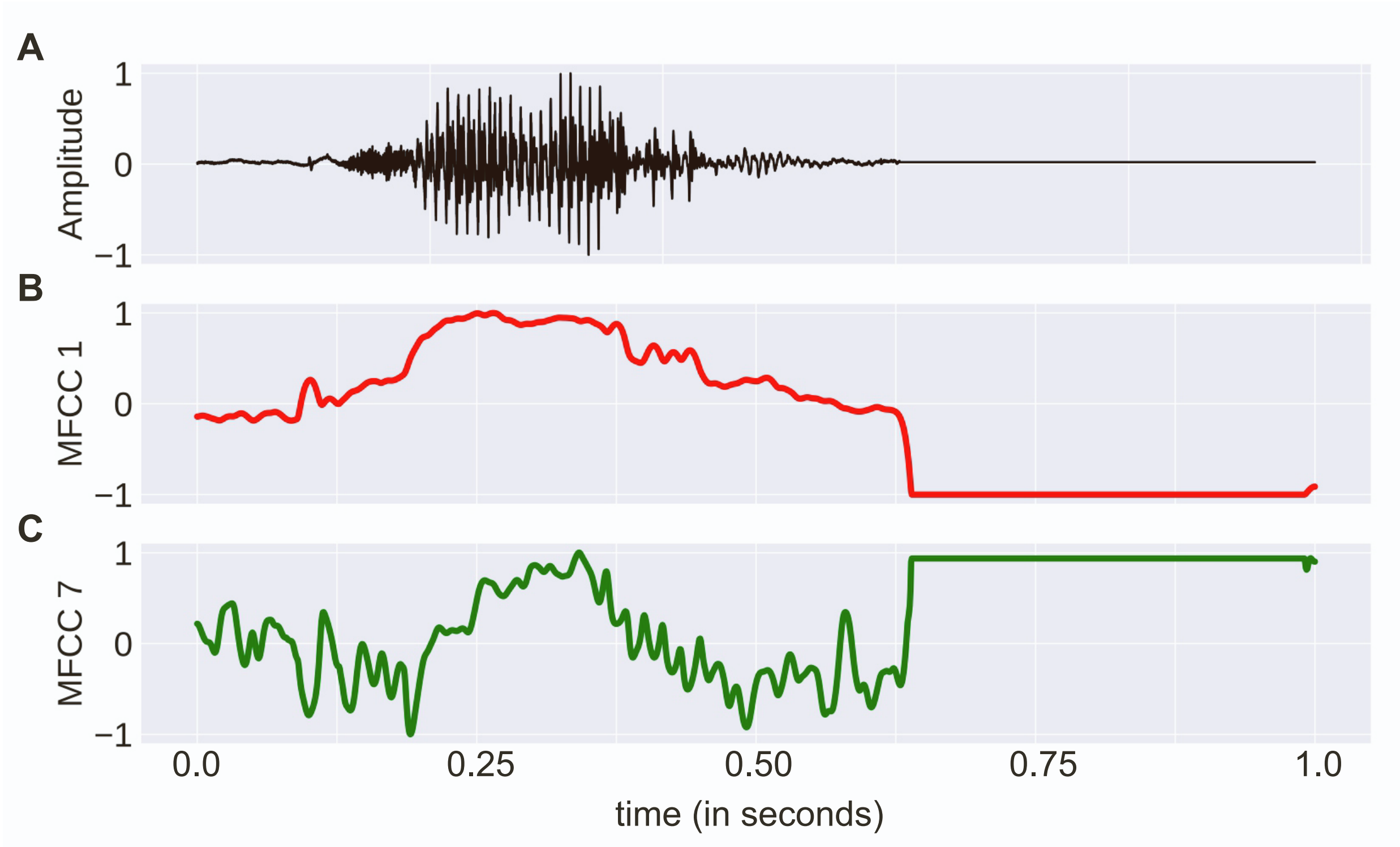

Figure S1: Mel-frequency cepstral coefficients (MFCCs) extracted from raw speech waveform. **(A)** Waveform of the spoken word "zero." **(B)** First MFCC coefficient, which closely resembles the speech envelope. **(C)** Seventh MFCC coefficient, which reflects finer spectral details. All MFCCs were normalized to [-1, 1] for comparison with the raw waveform.

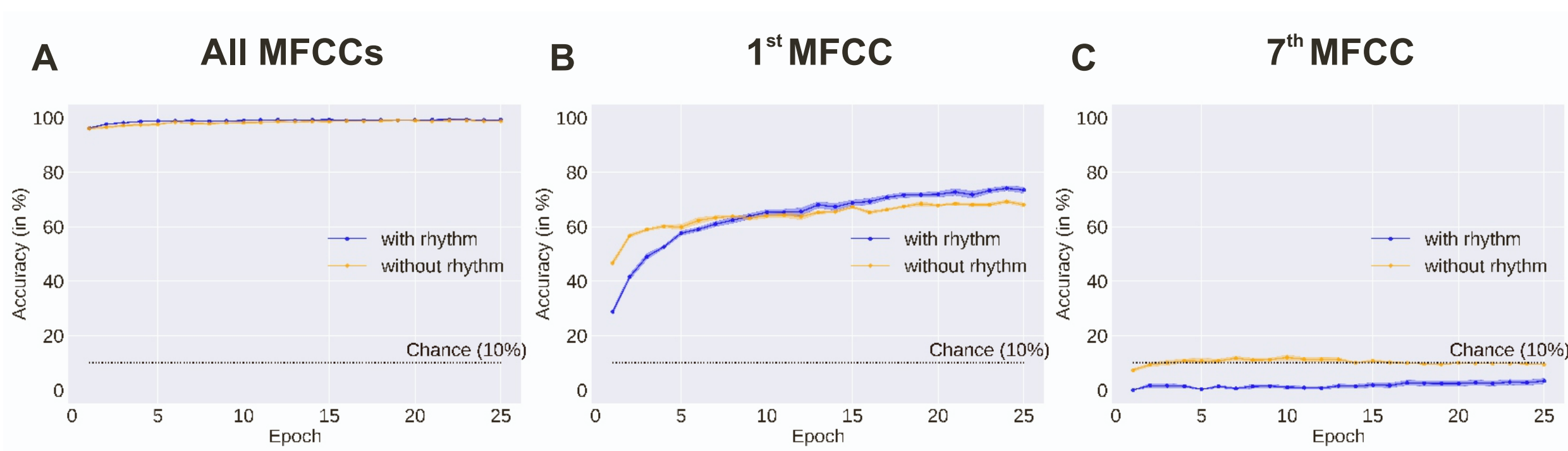

Figure S2: Performance with mel-frequency cepstral coefficients (MFCCs). Test accuracy per epoch of networks configured with frequency coefficients chosen uniformly at random from the range [1 Hz, 3 Hz] (blue). Values are averaged across ten random seeds. Shaded region depicts standard error. For reference, performance of the same network without endogenous oscillations is shown in yellow. Mel coefficients were **(A)** All, **(B)** 1st, **(C)** 7th.

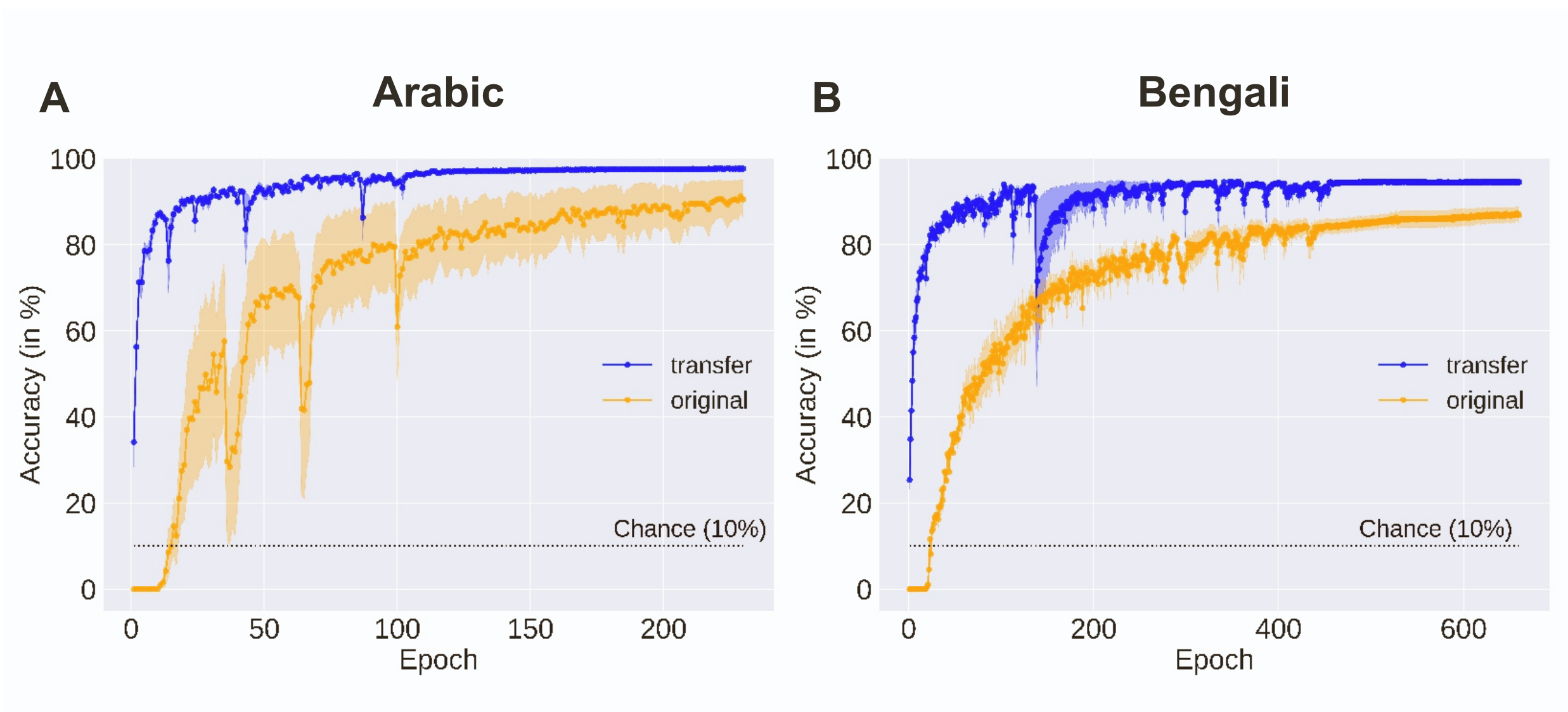

Figure S3: Transfer learning across languages. **(A)** Test accuracy per epoch for Arabic spoken digit recognition, comparing a network initialized with the parameters of a pre-trained English model (blue) to a network trained from scratch (yellow). **(B)** Same setup for Bengali spoken digit recognition. In both cases, the pre-trained network reaches near-perfect accuracy within a few epochs, whereas training from scratch requires over 100 epochs. Values are averaged across three random seeds. Shaded regions depict standard error.

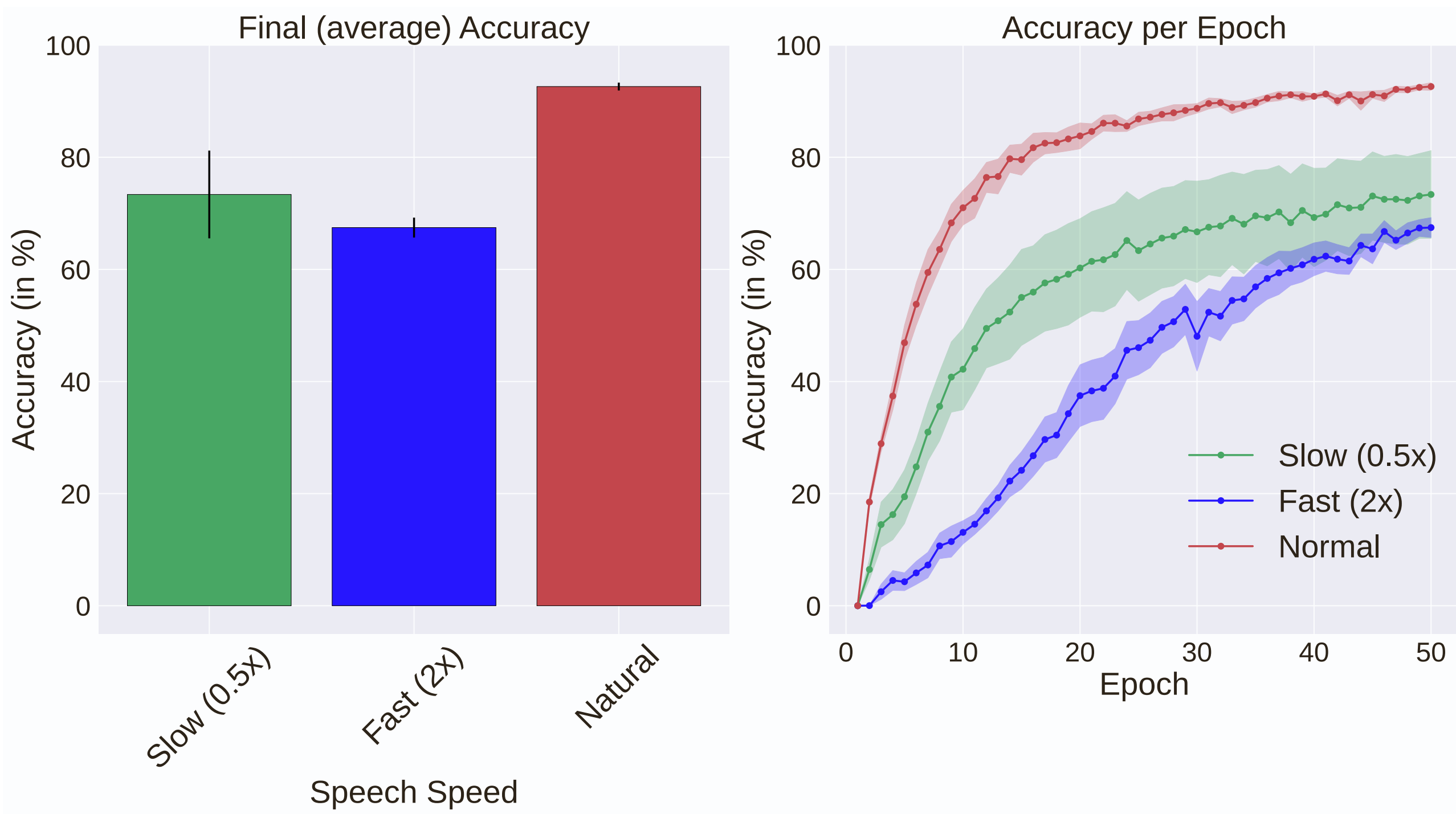

Figure S4: Effect of speech rate manipulation on performance. *Left:* Test accuracy of networks configured with [1 Hz, 5 Hz] frequency coefficients when the speech signal is slowed (0.5x), sped up (2x), and natural. *Right:* Test accuracy per epoch for the same networks. Values are averaged across ten random seeds. The shaded region depicts standard error.

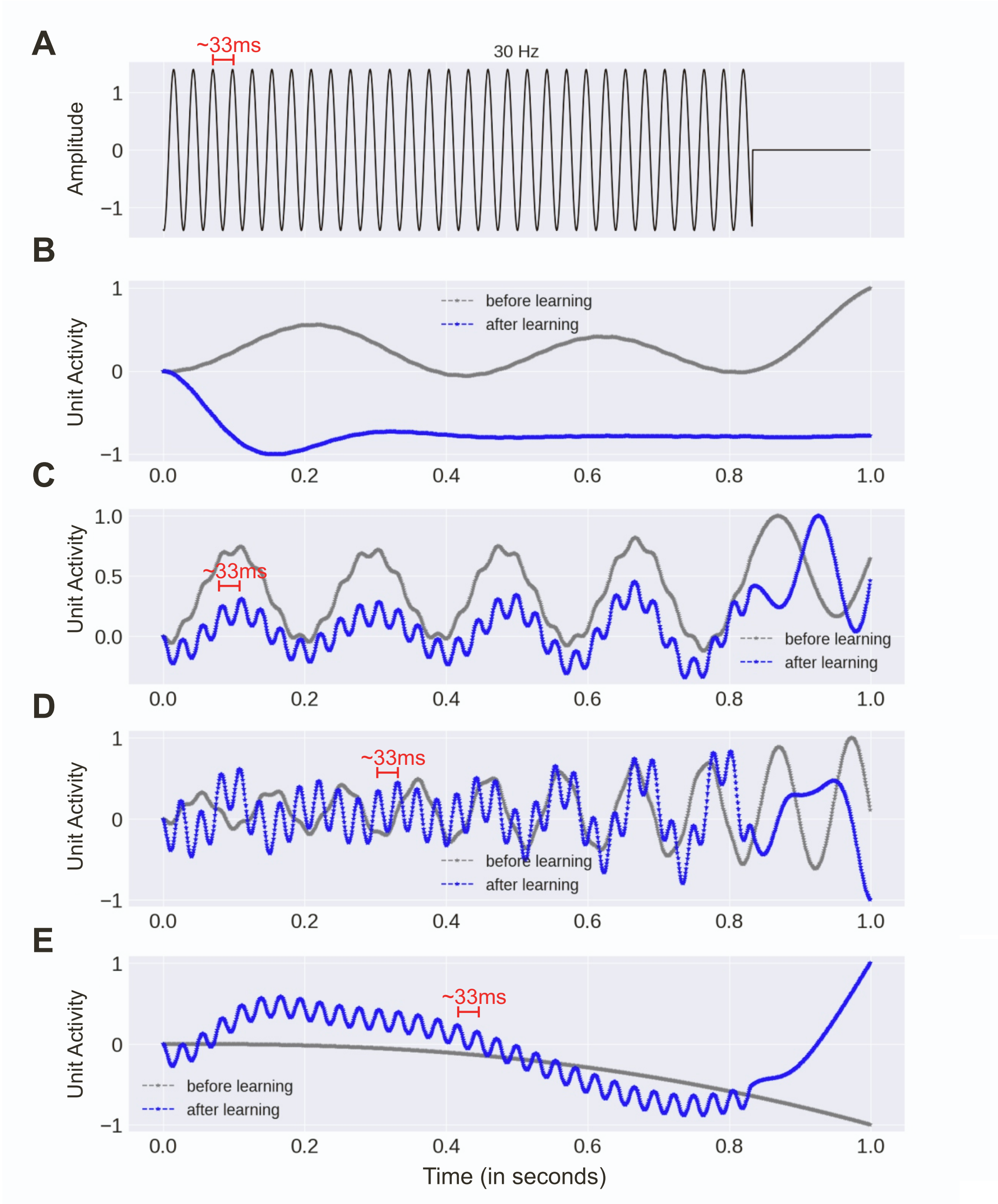

Figure S5: Network oscillations in response to a 30 Hz sinusoidal input. **(A)** Input waveform. **(B)** Response of an oscillator in a network configured with frequency coefficients drawn uniformly at random from the range [1 Hz, 10 Hz], **(C)** [10 Hz, 100 Hz], **(D)** [100 Hz, 110 Hz]. **(E)** Response when the frequency coefficients are set to 0. Oscillator activity is plotted as its normalized amplitude (scaled to [-1,1]). All networks, except for the network in **B**, tracks the temporal modulations of the input and correctly classifies the signal.

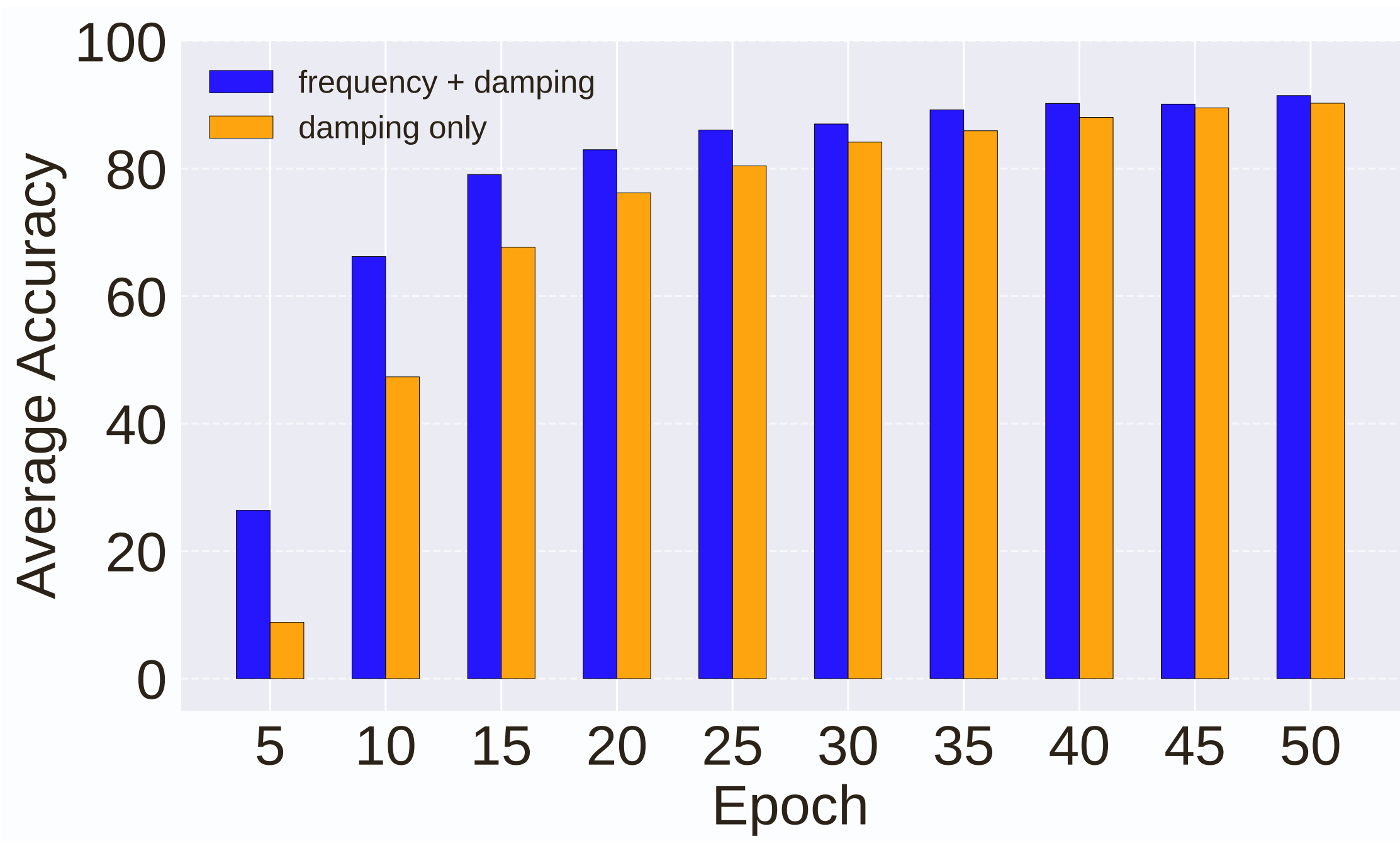

Figure S6: Task accuracy with damping only. Blue: Test accuracy on AudioMNIST (after every five epochs of training) of a network configured with frequency and damping coefficients drawn uniformly at random from the range [1 Hz, 3 Hz] and [0.1, 80], respectively. Yellow: same as blue, but with frequency coefficients set to 0. This network learns at a slower pace but eventually reaches high accuracy once it learns to oscillate with the speech envelope. Performances shown are averaged cross three random seeds.

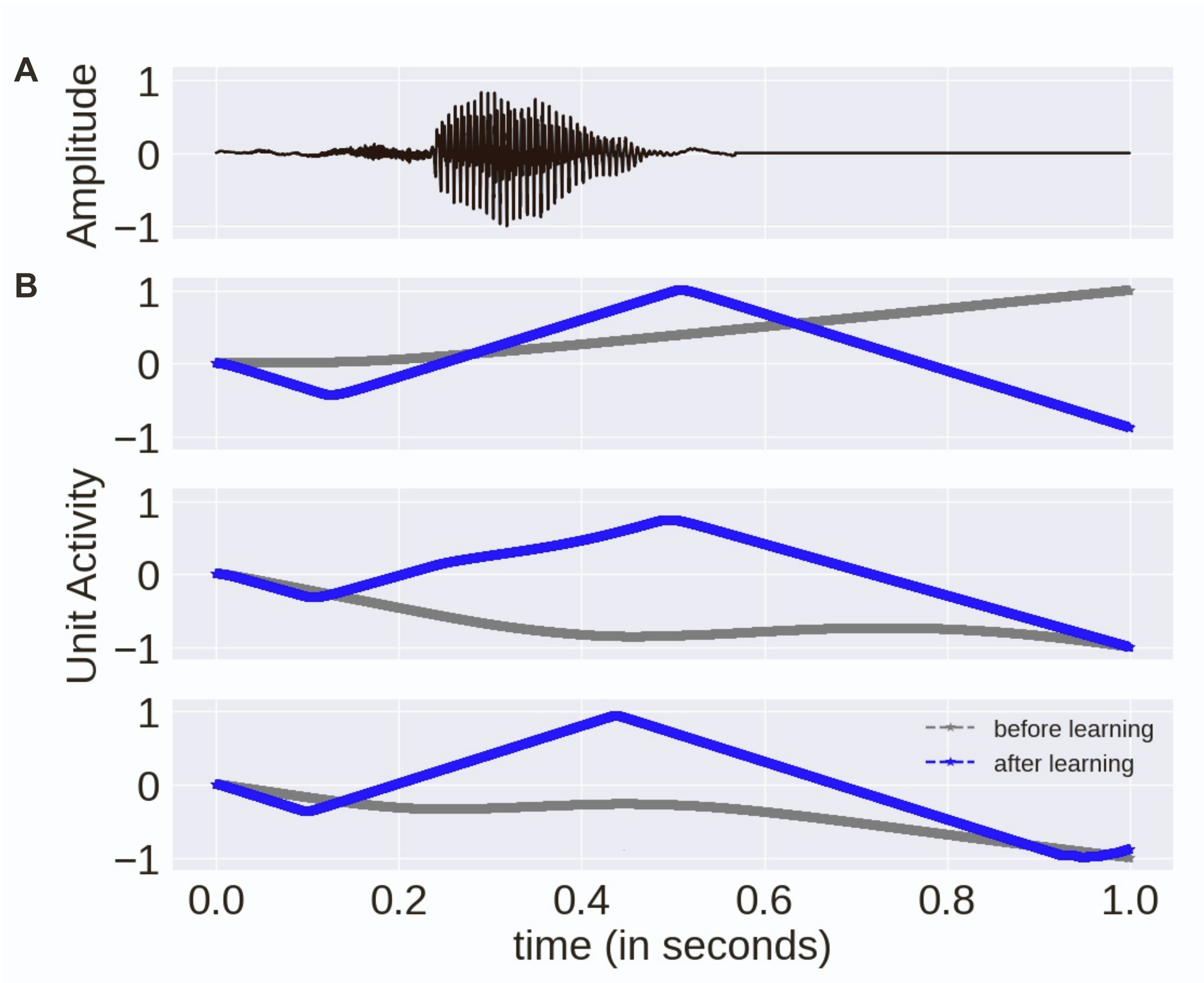

Figure S7: Oscillations in damped networks. **(A)** Waveform of the spoken word "three." **(B)** Example response of three network oscillators before (grey) and after training (blue) on AudioMNIST. The frequency coefficient of the network is set to 0 and the damping coefficients are drawn uniformly at random from the range [0.1, 80]. Before training, no clear oscillatory pattern is present. After training, the oscillators exhibit rhythmic activity at approximately 1 Hz, matching the approximately 1 Hz rhythm of the speech envelope. The network classified the input correctly after training. Oscillator activity is plotted as its normalized amplitude (scaled to [-1, 1]).

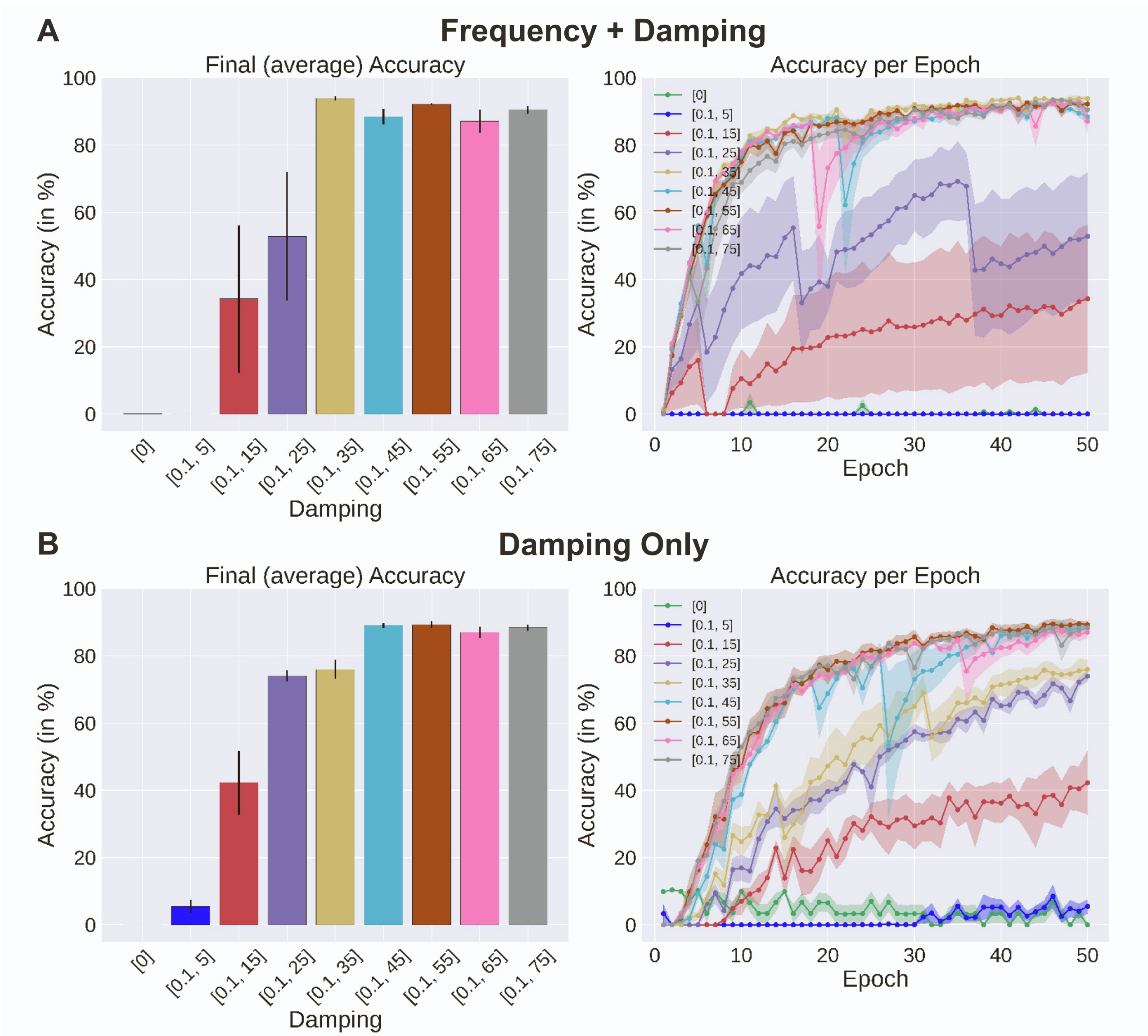

Figure S8: Impact of damping coefficient on network performance. **(A)** Accuracy in detecting English spoken words. Left: Test accuracy of networks configured with damping coefficients drawn uniformly at random from the specified ranges and oscillator frequencies drawn from the range $[1, 3]$ Hz. Values are averaged across three random seeds. Error bars depict standard error. Right: Test accuracy after every epoch of training. **(B)** Performance of networks with the same damping coefficients but with the frequency coefficient set to 0, illustrating a similar dependence on damping.

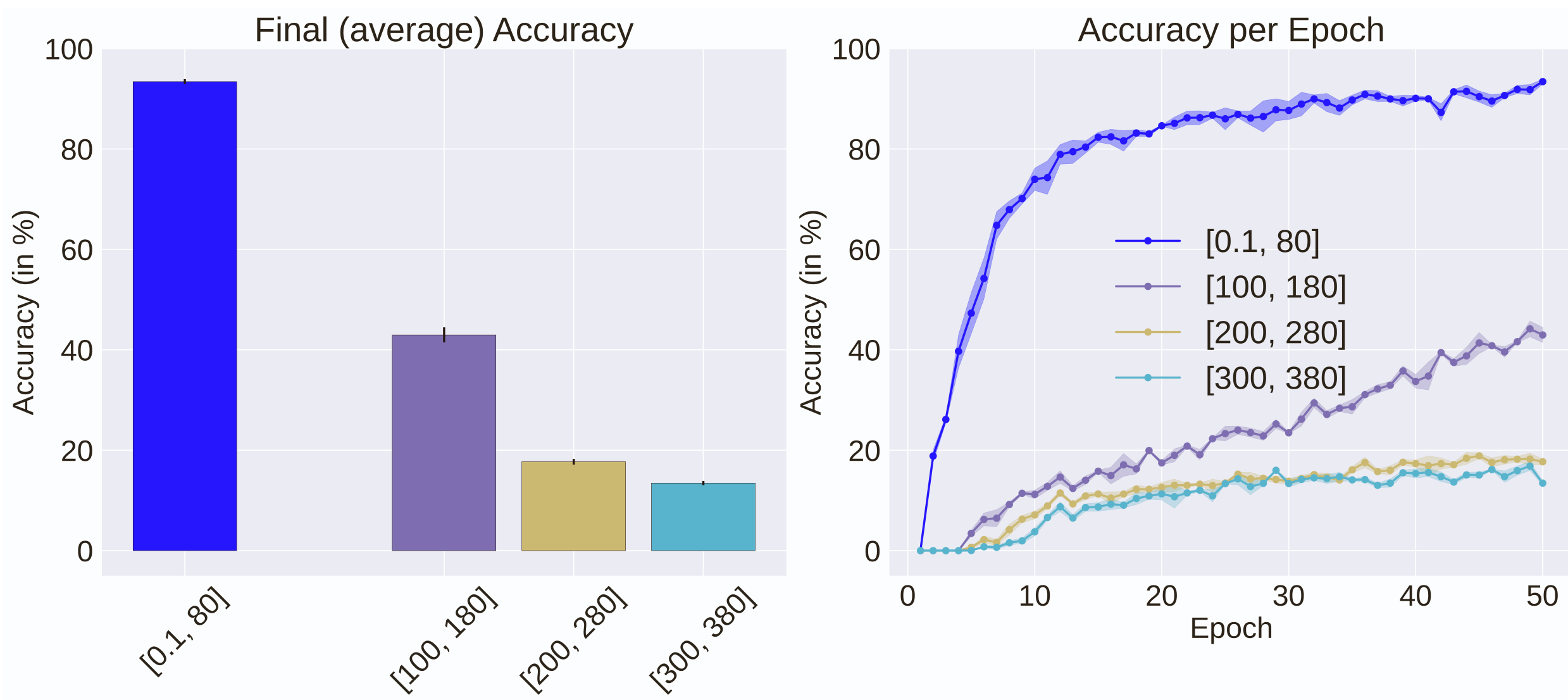

Figure S9: Impact of high damping coefficient on network performance. Accuracy in detecting English spoken words. Left: Test accuracy of networks configured with damping coefficients drawn uniformly at random from the specified ranges and oscillator frequencies drawn from the range $[1, 3]$ Hz. Values are averaged across three random seeds. Error bars depict standard error. Right: Test accuracy after every epoch of training. Overdamping the network substantially reduces performance.

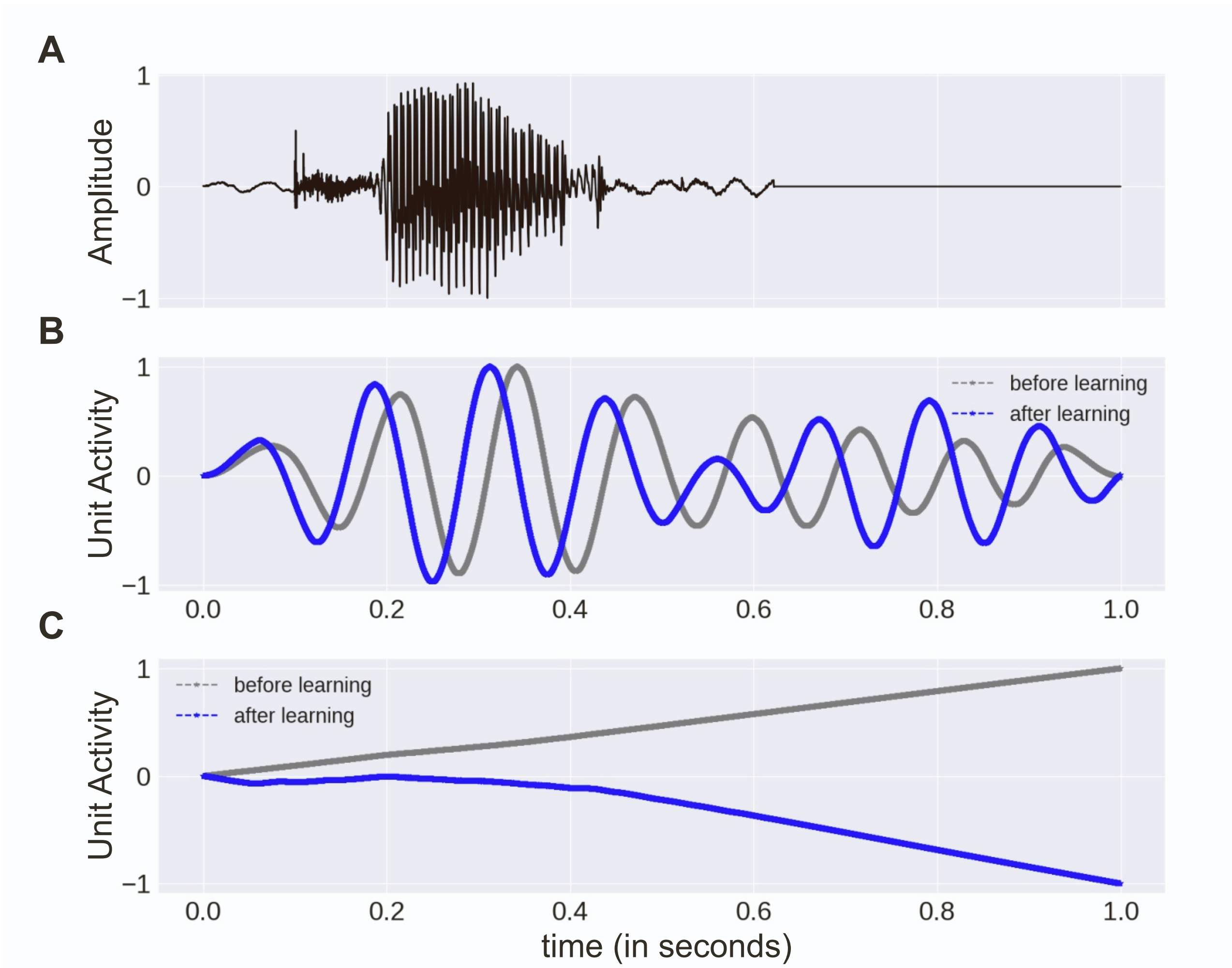

Figure S10: Network oscillations as a result of no damping and excessive damping. **(A)** Waveform of the spoken word "two" from the AudioMNIST dataset. **(B)** Example response of an oscillator in a network with damping coefficients set to 0 and frequency coefficients drawn from the range [1, 3] Hz. **(C)** Example response of an oscillator in a network with very high damping ($\epsilon = 240$) and frequency coefficients drawn from the range [1 Hz, 3 Hz]. Oscillator activity is plotted as its normalized amplitude (scaled to [-1,1]). Both networks classified the input incorrectly after training on the full dataset.
